# Comprehensive Analysis of Sulfated Flavonoids in *Eclipta prostrata* for Quality Evaluation

**DOI:** 10.3390/molecules29204888

**Published:** 2024-10-15

**Authors:** Ryunosuke Sato, Yuto Nishidono, Ken Tanaka

**Affiliations:** 1 College of Pharmaceutical Sciences, Ritsumeikan University, Kusatsu 525-8577, Shiga, Japan; ph0150vi@ed.ritsumei.ac.jp (R.S.); nisidono@fc.ristumei.ac.jp (Y.N.); 2 Research Organization of Science and Technology, Ritsumeikan University, Kusatsu 525-8577, Shiga, Japan

**Keywords:** *Eclipta prostrata*, sulfated flavonoids, LC–MS

## Abstract

*Eclipta prostrata* (Asteraceae) is employed as a hemostatic agent in many traditional medicines, owing to its sulfated flavonoid content. In this study, we obtained crude drug samples from three provinces collected in different years and analyzed their sulfated flavonoid contents using liquid chromatography–mass spectrometry (LC–MS) for quality evaluation. Because sulfated flavonoids are unstable and difficult to isolate from extracts, this study first synthesized a variety of sulfated flavonoids and accumulated spectral data in order to identify the compounds in *E. prostrata*. The LC–MS analysis of six crude drug samples revealed the presence of luteolin 7-sulfate, apigenin 7-sulfate, diosmetin 7-sulfate, and diosmetin 3′-sulfate. The samples without luteolin 3′-sulfate featured high apigenin 7-sulfate content. Although the samples were collected from the same locality, their compositions differed depending on the year of collection. Further, they were classified according to three patterns: (1) samples with luteolin 7-sulfate as the main component, (2) samples with apigenin 7-sulfate as the main component, and (3) samples with relatively high diosmetin sulfate content. Luteolin 7-sulfate typically exhibits relatively high erythrocyte aggregation efficiency and fibrinogen aggregation rate. These results demonstrate that the analysis of sulfated flavonoids is beneficial for the quality evaluation of *E. prostrata* for hemostatic applications.

## 1. Introduction

*Eclipta prostrata* (L.) L. (Asteraceae) is an annual plant found in tropical and subtropical regions globally. This plant have been used as a traditional medicine by many ethnic groups. The *Compendium of Materia Medica*, edited by Li Shizhen and published in China in the late 16th century, describes the benefits of the plant for hemostasis, dental treatment, and hair growth [1]. In India, this plant is used as an ayurvedic drug for revitalization and to treat aging. Additionally, it is widely used to treat skin diseases, jaundice and other liver diseases, gastrointestinal diseases, asthma and other respiratory diseases, fever, hair loss and whitening, cuts, and enlarged spleen [2,3]. The medicinal properties of the different parts of this plant vary; the juice of its leaves is mixed with honey to treat catarrh in infants, whereas its shoot’s juice mixed with mustard oil is used to treat diarrhea and dysentery [1,2,3]. Additionally, the entire plant is used to treat hepatitis, itching, hemoptysis, bleeding, hematochezia, hematuria, diarrhea, and diphtheria. Feng et al. summarized the plant’s traditional uses in other countries [3].

Regarding the chemical constituents of the plant, several compounds have been identified, including coumestane derivatives, triterpene saponins, and flavonoids [1,3,4,5,6]. Among the coumestane derivatives, wedelolactone has been reported to exhibit various biological activities, including selective and irreversible kinase inhibitory activity [7,8]. The *Chinese Pharmacopoeia* specifies wedelolactone as an indicator that assess the quality of *E. prostrata* [9]. Moreover, several sulfated flavonoids have been identified, and Lee et al. reported the isolation of luteolin-7-sulfate and apigenin-7-sulfate from *E. prostrata* [5]. Recently, He et al. reported that sulfated flavonoids generally show procoagulant activities under acidic conditions [10]. Considering these activities of the sulfated flavonoid components and the typical pharmacological activity (i.e., hemostasis) of this plant, it is necessary to consider the pattern of sulfated flavonoid components when assessing the quality of *E. prostrata*. However, sulfated flavonoids are unstable and difficult to isolate from plant extracts for structural analysis. Furthermore, most sulfated flavonoids are difficult to identify through an analysis of the mixtures, such as liquid chromatography–mass spectrometry (LC–MS), due to the lack of spectral data in the literature and the same molecular weight in the case of regioisomers. Therefore, in order to reliably identify sulfated flavonoids in the extract, it is necessary to synthesize possible target compounds.

In this study, we first synthesized a variety of sulfated flavonoids and accumulated extensive spectral data in order to identify the compounds in *E. prostrata* in detail. Additionally, various crude drugs derived from *E. prostrata* were collected, and their methanol extracts were analyzed using LC–MS to determine the patterns of their sulfated flavonoid contents for quality evaluation.

## 2. Results and Discussion

### 2.1. Preparation of Sulfated Flavonoids

**Figure 1:**
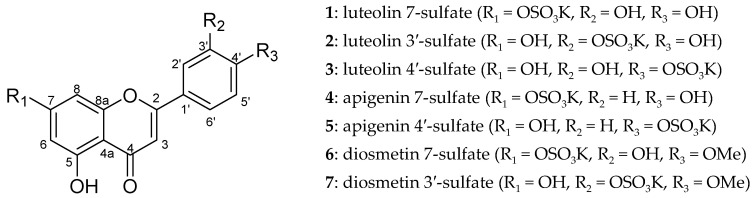
Structures of sulfated flavonoids.

Thus far, several attempts to prepare sulfated flavonoids have been reported [11,12]. Barron et al. reported the sulfation of flavonoids using dicylohexylcarbodiimide (DCC) and tetrabutylammonium hydrogen sulfate [13]. Additionally, Hayasaka et al. described the reaction with sulfur trioxide pyridine complexes [14]. In both reactions, there was no regioselectivity in sulfation, and each hydroxyl group in the flavonoid was sulfated to form a mixture. However, in the reaction with sulfur trioxide pyridine complexes, lower concentrations of 7-sulfate were formed, and in the reaction with DCC and tetrabutylammonium hydrogen sulfate, higher concentrations of disubstituted compounds were formed. Furthermore, the sulfated flavonoids were highly unstable unless in the form of sulfates, making the mixture difficult to separate. The reaction of DCC with tetrabutylammonium hydrogen sulfate produced a mixture of tetrabutylammonium and potassium salts in the post-treatment step. This required conversion to only the potassium salt, which reduced the final yield. Therefore, by replacing tetrabutylammonium hydrogen sulfate with potassium hydrogen sulfate, the formation of disubstituted products were suppressed and the salt-exchange process was omitted (Figure 1). Nuclear magnetic resonance (NMR) data for the prepared compounds are shown in Table 1 and Table 2 (raw NMR, ultraviolet (UV) spectral data, and UV data measured with an HPLC-PDA detector are shown in the Supplementary Materials).

**Figure 2:**
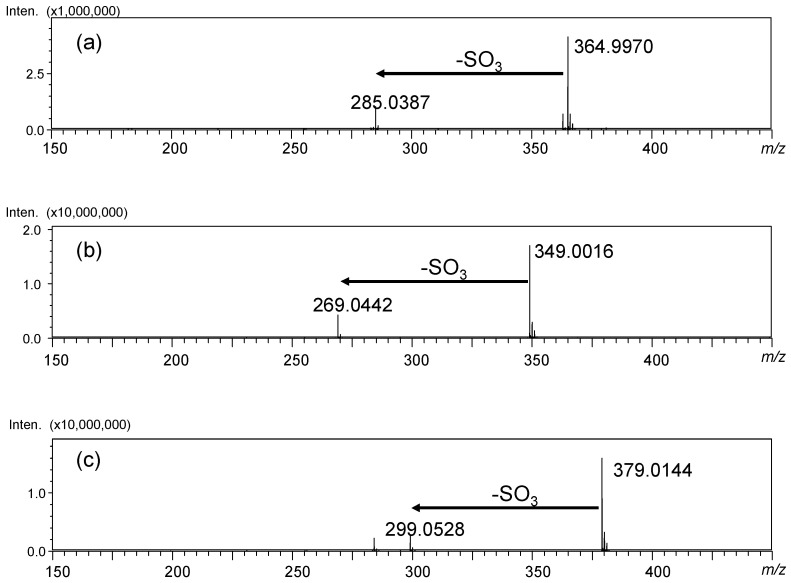
LC–MS spectra of (**a**) luteolin 7-sulfate (**1**), (**b**) apigenin 7-sulfate (**4**), and (**c**) diosmetin 7-sulfate (**6**).

In Figure 2, the LC–MS spectra of luteolin 7-sulfate (**1**), apigenin 7-sulfate (**4**), and diosmetin 7-sulfate (**6**) are shown. The characteristic fragmentation of the sulfated flavonoids is the desorption of SO_3_ to distribute fragment ions derived from the original flavonoid, which was also detected in all synthetic sulfated flavonoids (the MS spectra of the compounds and MS/MS spectra from respective (M–H)^−^ ions are shown in the Supplementary Materials) [15].

**Figure 3:**
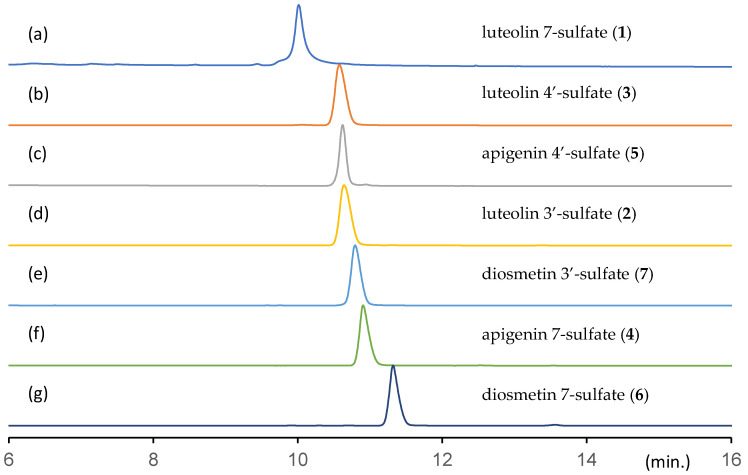
High-performance liquid chromatogram of sulfated flavonoids. Peak detection was performed with ultraviolet irradiation at 330 nm. (**a**) Luteolin 7-sulfate (**1**), (**b**) luteolin 4′-sulfate (**3**), (**c**) apigenin 4′-sulfate (**5**), (**d**) luteolin 3′-sulfate (**2**), (**e**) diosmetin 3′-sulfate (**7**), (**f**) apigenin 7-sulfate (**4**), and (**g**) diosmetin 7-sulfate (**6**).

In Figure 3, the high-performance liquid (HPLC) chromatograms of the synthesized sulfated flavonoids are shown. Under the HPLC analytical conditions used in this study, the compounds within the samples were separated, except for apigenin 4′-sulfate (**5**) and luteolin 3′-sulfate (**2**). Apigenin 4′-sulfate (**5**) and luteolin 3′-sulfate (**2**) were distinguished using mass chromatography because of their different molecular weights, which did not interfere with the analysis of the extracts from the *E. prostrata* specimens.

### 2.2. LC–MS Analysis of the Extracts of the E. prostrata Specimens

Six crude drug specimens of *E. prostrata* included Henan Province productions in 2019 (sample 1), 2020 (sample 2), 2021 (sample 3), and year of production unknown (sample 4); Hunan Province production in 2021 (sample 5); and Jiangxi Province production (year of production unknown, sample 6). These specimens were obtained from the main *E. prostrata* production areas in China and are representative of the crude drugs derived from *E. prostrata* distributed in China.

**Figure 4:**
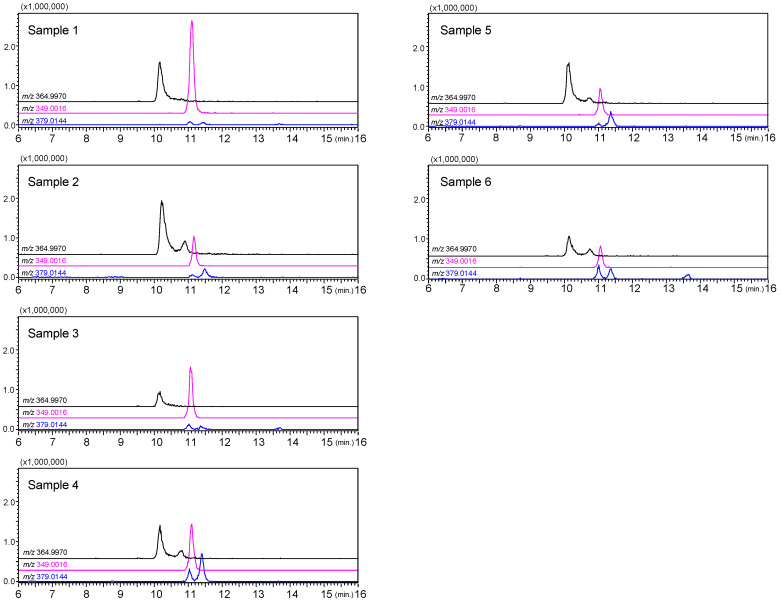
Liquid chromatography–mass spectrometry mass chromatograms of the extracts of the *E. prostrata* specimens: monitored by (M–H)^−^ ions of luteolin sulfate (*m*/*z* 364.9970), apigenin sulfate (*m*/*z* 349.0016) and diosmetin sulfate (*m*/*z* 379.0144).

**Figure 5:**
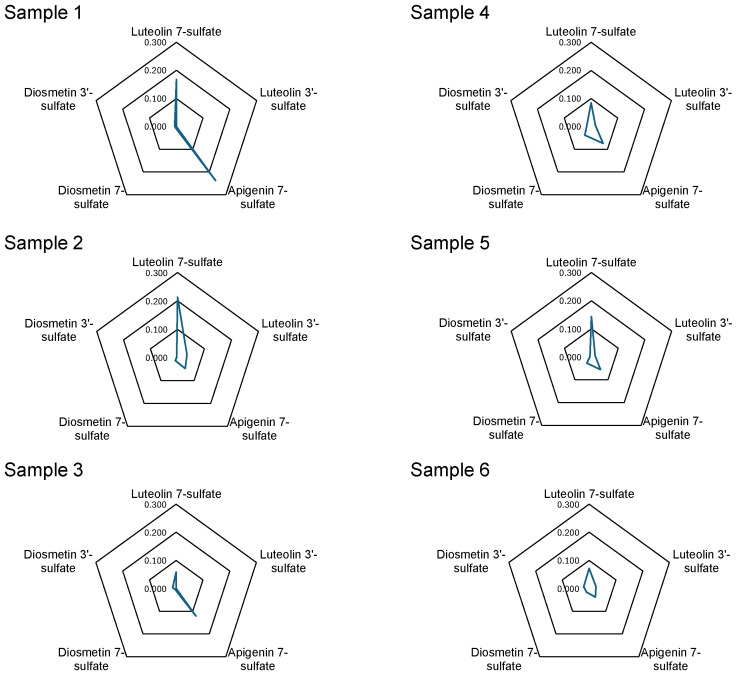
Radar diagram of the quantitative values shown in Table 3.

Mass chromatograms monitored by (M–H)^−^ ions of luteolin sulfate (*m*/*z* 364.9970), apigenin sulfate (*m*/*z* 349.0016), and diosmetin sulfate (*m*/*z* 379.0144) of the methanol extracts of six samples of *E. prostrata* is shown in Figure 4. (The total-ion chromatograms from the methanol extracts of six *E. prostrata* samples and 3D-HPLC chromatograms are shown in the Supplementary Materials). Luteolin 7-sulfate (**1**), apigenin 7-sulfate (**4**), diosmetin 7-sulfate (**6**), and diosmetin 3′-sulfate (**7**) were detected in all samples. Additionally, trace amounts of luteolin 3′-sulfate were detected in samples 2 and 4–6. Samples 1 and 3, in which luteolin 3′-sulfate (**2**) was not detected, had high apigenin 7-sulfate (**4**) content. The results of the quantification of each compound detected are shown in Table 3, and the quantitative values are shown in a radar diagram in Figure 5.

Sulfated flavonoids are typically found in a limited number of plants, including those of Apiaceae, Asteraceae, Bixaceae, Dyreniaceae, Frankeniaceae, Malvaceae, and Verbenaceae [11,16]. Cytoplasmic sulfotransferase (SOT) is involved in the biosynthesis of sulfated flavonoids and acts through a position-specific mechanism [16,17]. For example, flavonol SOT from *Arabidopsis thaliana* has a high affinity for kaempferol and flavonol glycosides and transfers sulfate groups to hydroxyl groups at positions 3 or 7 [18,19]. Conversely, flavonol SOTs from *Flaveria bidentis* have been reported to produce 4′ and 3′ sulfate derivatives with high affinity for quercetin [18,20]. Furthermore, Varin et al. reported that the most common flavonoid sulfates are represented trough sulfation at positions 7 > 3′ > 4′ > 6 > 8 and 3 > 7 > 4′ > 3′ for flavones and flavonols, respectively [16,21]. In this study, the sulfated flavonoids in *E. prostrata* were mainly modified at position 7, with only a few modified at the 3′, and 4′-position; modification was not found in the sulfated flavones. Our results support the reports of Varin et al.

Although samples 1–4 were collected from the same locality (Henan Province), they were classified into the following three patterns of composition depending on the year of collection: (1) luteolin 7-sulfate (**1**) was the main component (sample 2), (2) apigenin 7-sulfate (**4**) was the main component (sample 1 and 3), and (3) relatively high diosmetin sulfate content (sample 4). The samples obtained from Hunan Province (sample 5) and Jiangxi Province (sample 6) showed a similar pattern to sample 4 from Henan Province with a high diosmetin sulfate content. Although the functional role of the flavonoid sulfates in plant cells and tissues is not clear, Teles et al. have shown that sulfated flavonoid biosynthesis may be an ecological adaptation to the environment in addition to regulating plant growth through influencing auxin transport [17]. Furthermore, a strong correlation has been shown between plants growing in mineral salt-rich watersheds and the biosynthesis of sulfated flavonoids [16]. *E. prostrata* is a common weed in paddy fields, and from an ecological perspective, its chemical composition may be influenced by precipitation. Therefore, we examined the correlation between precipitation in Henan, Hunan, and Jiangxi provinces from May to July during 2019–2021 and the sulfated flavonoid content patterns of the samples analyzed in this study. According to meteorological data from China’s National Bureau of Statistics, in Zhengzhou, a major city in Henan Province, May–July precipitation in 2019 and 2021 was 67% and 55% of the precipitation in the decade, respectively. Hunan and Jiangxi have more precipitation than Henan, with June precipitation alone equal to the May–July total for Henan. In Hunan Province from 2019 to 2021, precipitation from May to July in 2021 was about the same as the 10-year average. Additionally, in Jiangxi, the precipitation from May to July in 2021 and 2022 constituted 51% and 63% of the decade’s average, respectively. In comparison with rainfall data, it was observed that apigenin 7-sulfate tends to increase in Henan when rainfall is low (e.g., samples 1 and 3). It has been considered that the levels of apigenin 7-sulfate might be low in Hunan and Jiangxi due to the generally high amounts of rainfall (e.g., samples 5 and 6). Regarding diosmetin sulfate, there was a tendency for diosmetin 7-sulfate concentrations to increase when precipitation in the area was normal (e.g., samples 2 and 5).

He et al. reported that luteolin 7-sulfate potassium salt (**1**) exhibited higher erythrocyte agglutination efficiencies and fibrinogen flocculation rates than apigenin 7-sulfate potassium salt (**4**) [10]. In terms of the hemostatic effect of *E. prostrata*, the crude drug containing high concentrations of luteolin 7-sulfate (**1**) was more advantageous, and it is necessary to note the difference in the composition of sulfated flavonoids found in this study, even though the samples were collected from the same region.

## 3. Materials and Methods

### 3.1. Crude Drug Specimens and Reagents

Six crude drug specimens of *E. prostrata* were obtained from Matsuura Yakugyo Co., Ltd. (Nagoya, Japan). The specimens included Henan Province productions in 2019 (sample 1), 2020 (sample 2), 2021 (sample 3), and year of production unknown (sample 4); Hunan Province production in 2021 (sample 5); and Jiangxi Province production (year of production unknown, sample 6). All voucher samples were deposited in the Laboratory of Pharmacognosy, College of Pharmaceutical Sciences, Ritsumeikan University (Shiga, Japan).

Apigenin, luteolin, and diosmetin were purchased from Tokyo Chemical Industry Co., Ltd. (Tokyo, Japan). In addition to these flavonoids, other commercially available chemicals and solvents were used without further purification.

Reversed-phased column chromatography was performed with Universal Premium Column ODS-SM (30 mm, 3.0 × 20.0 cm, Yamazen Co. (Osaka, Japan)). Size exclusion chromatography (SEC) was performed using TOYOPEARL HW-40F (NACALAI TESQUE Co. (Kyoto, Japan)). Desalination was performed using a Sep-Pak Vac 6 cc C18 cartridge (Waters Co. (Milford, MA, USA)). Pre-coated thin layer chromatography (TLC) was performed on Silica gel 60 RP-18 F254S (Merck (Darmstadt, Germany)) and a Polyamide FM Plate (FUJIFILM Wako Pure Chemical Co. (Osaka, Japan)). The spots on the TLC plates were detected by UV irradiation.

Other analytical-grade chemicals and chromatographic solvents (LC–MS grade) were purchased from Fujifilm Wako Pure Chemical (Osaka, Japan).

### 3.2. Apparatus

NMR spectra were recorded using a JNM-ECZ500R/S1 (JEOL Ltd. (Tokyo, Japan)), which was operated at 500 MHz (^1^H) and 125 MHz (^13^C). Tetramethylsilane (internal standard of NMR spectrometry) and deuterated dimethyl sulfoxide were obtained from Euriso-Top (Saint-Aubin, France). UV spectra were recorded on a Shimadzu UV-1800 spectrometer (Shimadzu Co., Kyoto, Japan).

For the LC–MS analyses, we used the mass spectrometer Shimadzu LC–IT–TOF (Shimadzu, Kyoto, Japan) equipped with a Shimadzu SPD 20A photodiode array UV–VIS detector and an electrospray ionization (ESI) interface. The ESI parameters were as follows: source voltage, −3.5 kV in negative-ion mode; capillary temperature, 200 °C; and nebulizer gas flow rate, 1.5 L/min. We used the mass spectrometer in negative-ion modes and recorded the scans from 150 to 1500 *m*/*z*. The constituents of the extracts were separated using the ODS column Waters Atlantis T3 (2.1 × 150 mm, 5 μm, 40 °C), and the binary mobile phase consisted of (A) 5 mM CH_3_COONH_4_ in water and (B) CH_3_CN. The compounds were eluted under the following gradient conditions: 0–30 min, linear gradient from 10% to 100% B, and 30–40 min of isocratic solution at 100% B.

### 3.3. Extraction of Constituents from E. prostrata

The crude drugs were weighed and powdered using a TUBE-MILL 100 milling machine (Model C S004, IKA, Staufen, Baden-Württemberg, Germany). Then, 3 g of the crude drug powder were precisely weighed and extracted with 140 mL of methanol under reflux conditions for 40 min using the B-811 Extraction System (BÜCHI Labortechnik, Flawil, St. Gallen, Switzerland). The extract was dried by removing the solvent under reduced pressure and weighed and dissolved in methanol to make a 10 mg extract/mL solution, which was filtered through a 0.45-μm polytetrafluoroethylene membrane. The same extraction procedure was performed for all crude drug samples.

### 3.4. Synthesis of Sulfate Conjugates

To a stirred solution of flavonoid (0.6 mmol, 1 equiv.) in pyridine (3 mL), DCC (6.0 mmol, 10 equiv.) was added at 0 °C. After 10 min, the solution of potassium hydrogen sulfate (1.2 mmol, 2 equiv.) in pyridine (16 mL) was added dropwise to the above solution, and the resulting reaction mixture was stirred at 0 °C for 3 days. Subsequently, pyridine was removed under reduced pressure, and 12 mL of potassium acetate solution in methanol (0.1 M) was added. The precipitate was filtered off and rinsed using methanol. The filtrate was evaporated, and the residue was subjected to reversed-phase chromatography (methanol–water 40:60 (*v*/*v*)) to obtain the mixture of regioisomers. This mixture was repeatedly subjected to SEC (methanol–water 30:70 (*v*/*v*)) to separate the respective mono-sulfates.

The obtained pure mono-sulfate was dissolved in 5 mL of potassium acetate buffer (pH = 4.6) to protonate the ionized phenolic hydroxy group, and the solution was subjected to a Sep-Pak Vac 6 cc C18 cartridge (washed using 16 mL of water and eluted using 20 mL of methanol) to obtain the pure sulfate potassium salt.

Compounds were identified by the comparison of various spectral data with literature values [22] and the analysis of MS and NMR spectral data.

Luteolin 7-sulfate potassium salt (**1**): yellow solid; 6.7% yield; UV (MeOH) λmax (log ε): 254 (4.13), 266 (sh 4.08), 349 (4.20) nm; ^1^H and ^13^C NMR, see Table 1 and Table 2.

Luteolin 3′-sulfate potassium salt (**2**): yellow solid; 9.3% yield; UV (MeOH) λmax (log ε): 269 (4.15), 334 (4.17) nm; ^1^H and ^13^C NMR, see Table 1 and Table 2.

Luteolin 4′-sulfate potassium salt (**3**): yellow solid; 9.3% yield; UV (MeOH) λmax (log ε): 269 (4.14), 329 (4.01) nm; ^1^H and ^13^C NMR, see Table 1 and Table 2.

Apigenin 7-sulfate potassium salt (**4**): pale yellow solid; 7.0% yield; UV (MeOH) λmax (log ε): 268 (4.17), 333 (4.26) nm; ^1^H and ^13^C NMR, see Table 1 and Table 2.

Apigenin 4′-sulfate potassium salt (**5**): pale yellow solid; 9.8% yield, UV (MeOH) λmax (log ε): 269 (4.34), 319 (4.17) nm; ^1^H and ^13^C NMR, see Table 1 and Table 2.

Diosmetin 7-sulfate potassium salt (**6**): yellow solid; 5.2% yield; UV (MeOH) λmax (log ε): 251 (4.05), 268 (4.00), 343 (4.09) nm; ^1^H and ^13^C NMR, see Table 1 and Table 2.

Diosmetin 3′-sulfate potassium salt (**7**): yellow solid; 6.2% yield; UV (MeOH) λmax (log ε): 269 (4.32), 330 (4.34) nm; ^1^H and ^13^C NMR, see Table 1 and Table 2.

## 4. Conclusions

We conducted a detailed analysis of the sulfated flavonoids contained in *E. prostrata*, traditionally used as a hemostatic agent. The use of DCC and potassium hydrogen sulfate in the synthesis of sulfated flavonoids, which is necessary for the accurate identification of plant contents, improved the composition of the reaction’s products and the efficiency of postprocessing. The LC–MS analysis of six crude drug samples from different regions collected in different years showed luteolin 7-sulfate (**1**), apigenin 7-sulfate (**4**), diosmetin 7-sulfate (**6**), and diosmetin 3′-sulfate (**7**) in all samples. Further, trace amounts of luteolin 3′-sulfate were detected in some samples. Samples in which luteolin 3′-sulfate (**2**) was not detected had relatively high apigenin 7-sulfate (**4**) content. Even though the samples were collected from the same locality (Henan Province), they had different compositions of sulfated flavonoids. Thus, they were classified into the following three groups based on the compositions and the year of collection: (1) samples with luteolin 7-sulfate (**1**) as the main component, (2) samples with apigenin 7-sulfate (**4**) as the main component, and (3) samples with relatively high diosmetin sulfate content. Moreover, luteolin 7-sulfate (**1**) typically exhibited a higher erythrocyte aggregation efficiency and fibrinogen aggregation rate than apigenin 7-sulfate (**4**). Thus, in terms of hemostatic applications, the results demonstrate that the analysis of sulfated flavonoids is valuable for evaluating the quality of *E. prostrata*.

## Figures and Tables

**Table 1:** ^1^H-nuclear magnetic resonance (NMR) data of sulfated flavonoids in deuterated dimethyl sulfoxide (DMSO-*d*_6_; δ *ppm*, (milt, *J* in Hz), s: singlet, d: doublet, dd: double doublet).

Position	Compound						
	1	2	3	4	5	6	7
3	6.73 (s)	6.69 (s)	6.78 (s)	6.83 (s)	6.85 (s)	6.81 (s)	6.66 (s)
6	6.51 (d, 1.9)	6.18 (d, 1.9)	6.19 (d, 1.9)	6.56 (d, 1.9)	6.20 (d, 1.9)	6.53 (d, 1.9)	6.19 (d, 2.3)
8	7.02 (d, 1.9)	6.46 (d, 1.9)	6.48 (d, 1.9)	7.01 (d, 1.9)	6.50 (d, 1.9)	7.03 (d, 1.9)	6.43 (d, 2.3)
2′	7.45 (d, 2.2)	7.88 (d, 2.3)	7.46 (d, 2.0)	7.95 (d, 8.8)	7.99 (d, 9.0)	7.47 (d, 2.3)	8.07 (d, 2.3)
3′	－	－	－	6.92 (d, 8.8)	7.34 (d, 9.0)	－	－
4′-OMe	－	－	－	－	－	3.86 (s)	3.84 (s)
5′	6.88 (d, 8.0)	6.97 (d, 8.6)	7.42 (d, 9.0)	6.92 (d, 8.8)	7.34 (d, 9.0)	7.08 (d, 8.8)	7.13 (d, 8.8)
6′	7.44 (dd, 2.2, 8.0)	7.68 (dd, 2.3, 8.6)	7.47 (d, 2.0, 9.0)	7.95 (d, 8.8)	7.99 (d, 9.0)	7.56 (dd, 2.3, 8.8)	7.74 (dd, 2.3, 8.8)

**Table 2:** ^13^C-NMR data of the sulfated flavonoids in DMSO-*d*_6_ (δ *ppm*).

Position	Compound						
	1	2	3	4	5	6	7
2	164.3	163.3	163.2	164.2	163.3	163.9	163.4
3	103.0	103.2	104.2	102.9	104.0	103.6	103.5
4	182.0	181.6	181.7	182.0	181.7	182.0	181.5
5	160.5	161.4	161.4	160.4	161.4	159.6	161.4
6	102.1	98.8	98.8	102.2	98.8	102.1	98.9
7	159.5	164.1	164.2	159.5	164.2	160.4	164.2
8	97.6	93.8	93.9	97.7	94.0	97.6	93.8
4a	105.6	103.7	103.8	105.6	103.7	105.6	103.7
8a	156.3	157.2	157.3	156.3	157.3	156.3	157.2
1′	121.3	121.5	126.2	120.9	124.6	122.8	122.2
2′	113.3	120.6	114.5	128.5	127.5	112.9	118.6
3′	145.7	141.4	148.8	116.0	120.0	146.9	142.9
4′	149.8	152.9	144.6	161.3	156.9	151.2	153.8
4′-OMe	－	－	－	－	－	55.7	55.8
5′	116.1	117.5	122.1	116.3	120.0	112.2	112.7
6′	119.1	123.4	118.0	128.5	127.5	118.7	122.4

**Table 3:** Contents of sulfated flavonoids in the dry samples.

Compounds	Contents (% of Dry Plant)					
	Sample 1	Sample 2	Sample 3	Sample 4	Sample 5	Sample 6
Luteolin 7-sulfate (**1**)	0.168 ± 0.002	0.214 ± 0.024	0.059 ± 0.008	0.084 ± 0.013	0.144 ± 0.009	0.072 ± 0.007
Luteolin 3′-sulfate (**2**)	-	0.036 ± 0.005	-	0.016 ± 0.005	0.014 ± 0.005	0.026 ± 0.005
Apigenin 7-sulfate (**4**)	0.237 ± 0.011	0.047 ± 0.005	0.119 ± 0.008	0.074 ± 0.005	0.055 ± 0.008	0.037 ± 0.005
Diosmetin 7-sulfate (**6**)	0.004 ± 0.001	0.013 ± 0.002	0.005 ± 0.001	0.037 ± 0.005	0.027 ± 0.006	0.016 ± 0.003
Diosmetin 3′-sulfate (**7**)	0.006 ± 0.001	0.004 ± 0.002	0.013 ± 0.001	0.016 ± 0.001	0.005 ± 0.001	0.021 ± 0.003

## Data Availability

The data presented in this study are available on request from the corresponding author.

## Supplementary Materials

The following supporting information can be downloaded at: https://www.mdpi.com/article/10.3390/molecules29204888/s1, Figure S1: ^1^H NMR Spectrum of Luteolin 7-Sulfate Potassium Salt in DMSO-*d*_6_; Figure S2: ^13^C NMR Spectrum of Luteolin 7-Sulfate Potassium Salt in DMSO-*d*_6_; Figure S3: ^1^H NMR Spectrum of Luteolin 3′-Sulfate Potassium Salt in DMSO-*d*_6_; Figure S4: ^13^C NMR Spectrum of Luteolin 3′-Sulfate Potassium Salt in DMSO-*d*_6_; Figure S5: ^1^H NMR Spectrum of Luteolin 4′-Sulfate Potassium Salt in DMSO-*d*_6_; Figure S6: ^13^C NMR Spectrum of Luteolin 4′-Sulfate Potassium Salt in DMSO-*d*_6_; Figure S7: ^1^H NMR Spectrum of Apigenin 7-Sulfate Potassium Salt in DMSO-*d*_6_; Figure S8: ^3^C NMR Spectrum of Apigenin 7-Sulfate Potassium Salt in DMSO-*d*_6_; Figure S9: ^1^H NMR Spectrum of Apigenin 4′-Sulfate Potassium Salt in DMSO-*d*_6_; Figure S10: ^13^C NMR Spectrum of Apigenin 4′-Sulfate Potassium Salt in DMSO-*d*_6_; Figure S11: ^1^H NMR Spectrum of Diosmetin 7-Sulfate Potassium Salt in DMSO-*d*_6_; Figure S12: ^13^C NMR Spectrum of Diosmetin 7-Sulfate Potassium Salt in DMSO-*d*_6_; Figure S13: ^1^H NMR Spectrum of Diosmetin 3′-Sulfate Potassium Salt in DMSO-*d*_6_; Figure S14: ^13^C NMR Spectrum of Diosmetin 3′-Sulfate Potassium Salt in DMSO-*d*_6_; Figure S15: UV Spectrum of Luteolin 7-Sulfate Potassium Salt in MeOH; Figure S16: UV Spectrum of Luteolin 3′-Sulfate Potassium Salt in MeOH; Figure S17: UV Spectrum of Luteolin 4′-Sulfate Potassium Salt in MeOH; Figure S18: UV Spectrum of Apigenin 7-Sulfate Potassium Salt in MeOH; Figure S19: UV Spectrum of Apigenin 4′-Sulfate Potassium Salt in MeOH; Figure S20: UV Spectrum of Diosmetin 7-Sulfate Potassium Salt in MeOH; Figure S21: UV Spectrum of Diosmetin 3′-Sulfate Potassium Salt in MeOH; Figure S22: UV Spectrum of Luteolin 7-Sulfate Measured with an HPLC-PDA Detector; Figure S23: UV Spectrum of Luteolin 3′-Sulfate Measured with an HPLC-PDA Detector; Figure S24: UV Spectrum of Luteolin 4′-Sulfate Measured with an HPLC-PDA Detector; Figure S25: UV Spectrum of Apigenin 7-Sulfate Measured with an HPLC-PDA Detector; Figure S26: UV Spectrum of Apigenin 4′-Sulfate Measured with an HPLC-PDA Detector; Figure S27: UV Spectrum of Diosmetin 7-Sulfate Measured with an HPLC-PDA Detector; Figure S28: UV Spectrum of Diosmetin 3′-Sulfate Measured with an HPLC-PDA Detector; Figure S29: MS and MS/MS Spectra of Luteolin 7-Sulfate; Figure S30: MS and MS/MS Spectra of Luteolin 3′-Sulfate; Figure S31: MS and MS/MS Spectra of Luteolin 4′-Sulfate; Figure S32: MS and MS/MS Spectra of Apigenin 7-Sulfate; Figure S33: MS and MS/MS Spectra of Apigenin 4′-Sulfate; Figure S34: MS and MS/MS Spectra of Diosmetin 7-Sulfate; Figure S35: MS and MS/MS Spectra of Diosmetin 3′-Sulfate; Figure S36: Liquid Chromatography–Mass Spectrometry Total-ion Chromatograms and Mass Chromatograms of the Extracts of the *E. prostrata* Specimens; Figure S37: 3D-HPLC Chromatogram of Sample 1 (Henan Province, 2019); Figure S38: 3D-HPLC Chromatogram of Sample 2 (Henan Province, 2020); Figure S39: 3D-HPLC Chromatogram of Sample 3 (Henan Province, 2021); Figure S40: 3D-HPLC Chromatogram of Sample 4 (Henan Province); Figure S41: 3D-HPLC Chromatogram of Sample 5 (Hunan Province, 2021); Figure S42: 3D-HPLC Chromatogram of Sample 6 (Jiangxi Province).
