# Supplementary material for: Comprehensive Analysis of Sulfated Flavonoids in Eclipta prostrata for Quality Evaluation
Source: Molecules. 2024 Oct 15;29(20):4888. doi: 10.3390/molecules29204888 (PMC11509997; doi:10.3390/molecules29204888)
Supplement: Supplementary file 1 [file molecules-29-04888-s001.zip › molecules-3233860-supplementary.pdf]

Supporting Information

Comprehensive Analysis of Sulfated Flavonoids in *Eclipta prostrata* for Quality Evaluation

Ryunosuke Sato [1], Yuto Nishidono [1,2] and Ken Tanaka [1,*]

1 College of Pharmaceutical Sciences, Ritsumeikan University, Kusatsu 525-8577, Shiga, Japan; ph0150vi@ed.ritsumei.ac.jp (R.S.); nisidono@fc.ristumei.ac.jp (Y.N.)
2 Research Organization of Science and Technology, Ritsumeikan University, Kusatsu 525-8577, Shiga, Japan
* Correspondence: ktanaka@fc.ritsumei.ac.jp; Tel.: +81-77-561-5179

List of Contents

Figure S1. ^{1}H NMR Spectrum of Luteolin 7-Sulfate Potassium Salt in DMSO-d_6 3
Figure S2. ^{13}C NMR Spectrum of Luteolin 7-Sulfate Potassium Salt in DMSO-d_6 3
Figure S3. ^{1}H NMR Spectrum of Luteolin 3'-Sulfate Potassium Salt in DMSO-d_6 4
Figure S4. ^{13}C NMR Spectrum of Luteolin 3'-Sulfate Potassium Salt in DMSO-d_6 4
Figure S5. ^{1}H NMR Spectrum of Luteolin 4'-Sulfate Potassium Salt in DMSO-d_6 5
Figure S6. ^{13}C NMR Spectrum of Luteolin 4'-Sulfate Potassium Salt in DMSO-d_6 5
Figure S7. ^{1}H NMR Spectrum of Apigenin 7-Sulfate Potassium Salt in DMSO-d_6 6
Figure S8. ^{3}C NMR Spectrum of Apigenin 7-Sulfate Potassium Salt in DMSO-d_6 6
Figure S9. ^{1}H NMR Spectrum of Apigenin 4'-Sulfate Potassium Salt in DMSO-d_6 7
Figure S10. ^{13}C NMR Spectrum of Apigenin 4'-Sulfate Potassium Salt in DMSO-d_6 7
Figure S11. ^{1}H NMR Spectrum of Diosmetin 7-Sulfate Potassium Salt in DMSO-d_6 8
Figure S12. ^{13}C NMR Spectrum of Diosmetin 7-Sulfate Potassium Salt in DMSO-d_6 8
Figure S13. ^{1}H NMR Spectrum of Diosmetin 3'-Sulfate Potassium Salt in DMSO-d_6 9
Figure S14. ^{13}C NMR Spectrum of Diosmetin 3'-Sulfate Potassium Salt in DMSO-d_6 9
Figure S15. UV Spectrum of Luteolin 7-Sulfate Potassium Salt in MeOH 10
Figure S16. UV Spectrum of Luteolin 3'-Sulfate Potassium Salt in MeOH 10
Figure S17. UV Spectrum of Luteolin 4'-Sulfate Potassium Salt in MeOH 10
Figure S18. UV Spectrum of Apigenin 7-Sulfate Potassium Salt in MeOH 11
Figure S19. UV Spectrum of Apigenin 4'-Sulfate Potassium Salt in MeOH 11
Figure S20. UV Spectrum of Diosmetin 7-Sulfate Potassium Salt in MeOH 11
Figure S21. UV Spectrum of Diosmetin 3'-Sulfate Potassium Salt in MeOH 12
Figure S22. UV Spectrum of Luteolin 7-Sulfate Measured with an HPLC-PDA Detector 12
Figure S23. UV Spectrum of Luteolin 3'-Sulfate Measured with an HPLC-PDA Detector 13
Figure S24. UV Spectrum of Luteolin 4'-Sulfate Measured with an HPLC-PDA Detector 13
Figure S25. UV Spectrum of Apigenin 7-Sulfate Measured with an HPLC-PDA Detector 14
Figure S26. UV Spectrum of Apigenin 4'-Sulfate Measured with an HPLC-PDA Detector 14
Figure S27. UV Spectrum of Diosmetin 7-Sulfate Measured with an HPLC-PDA Detector 15
Figure S28. UV Spectrum of Diosmetin 3'-Sulfate Measured with an HPLC-PDA Detector 15
Figure S29. MS and MS/MS Spectra of Luteolin 7-Sulfate 16
Figure S30. MS and MS/MS Spectra of Luteolin 3'-Sulfate 16
Figure S31. MS and MS/MS Spectra of Luteolin 4'-Sulfate 17
Figure S32. MS and MS/MS Spectra of Apigenin 7-Sulfate 17
Figure S33. MS and MS/MS Spectra of Apigenin 4'-Sulfate 18
Figure S34. MS and MS/MS Spectra of Diosmetin 7-Sulfate 18
Figure S35. MS and MS/MS Spectra of Diosmetin 3'-Sulfate 19

Figure S36. Liquid Chromatography–Mass Spectrometry Total-ion Chromatograms and Mass Chromatograms of the Extracts of the *E. prostrata* Specimens 19
Figure S37. 3D-HPLC Chromatogram of Sample 1 (Henan Province, 2019) 20
Figure S38. 3D-HPLC Chromatogram of Sample 2 (Henan Province, 2020) 20
Figure S39. 3D-HPLC Chromatogram of Sample 3 (Henan Province, 2021) 21
Figure S40. 3D-HPLC Chromatogram of Sample 4 (Henan Province) 21
Figure S41. 3D-HPLC Chromatogram of Sample 5 (Hunan Province, 2021) 22
Figure S42. 3D-HPLC Chromatogram of Sample 6 (Jiangxi Province) 22

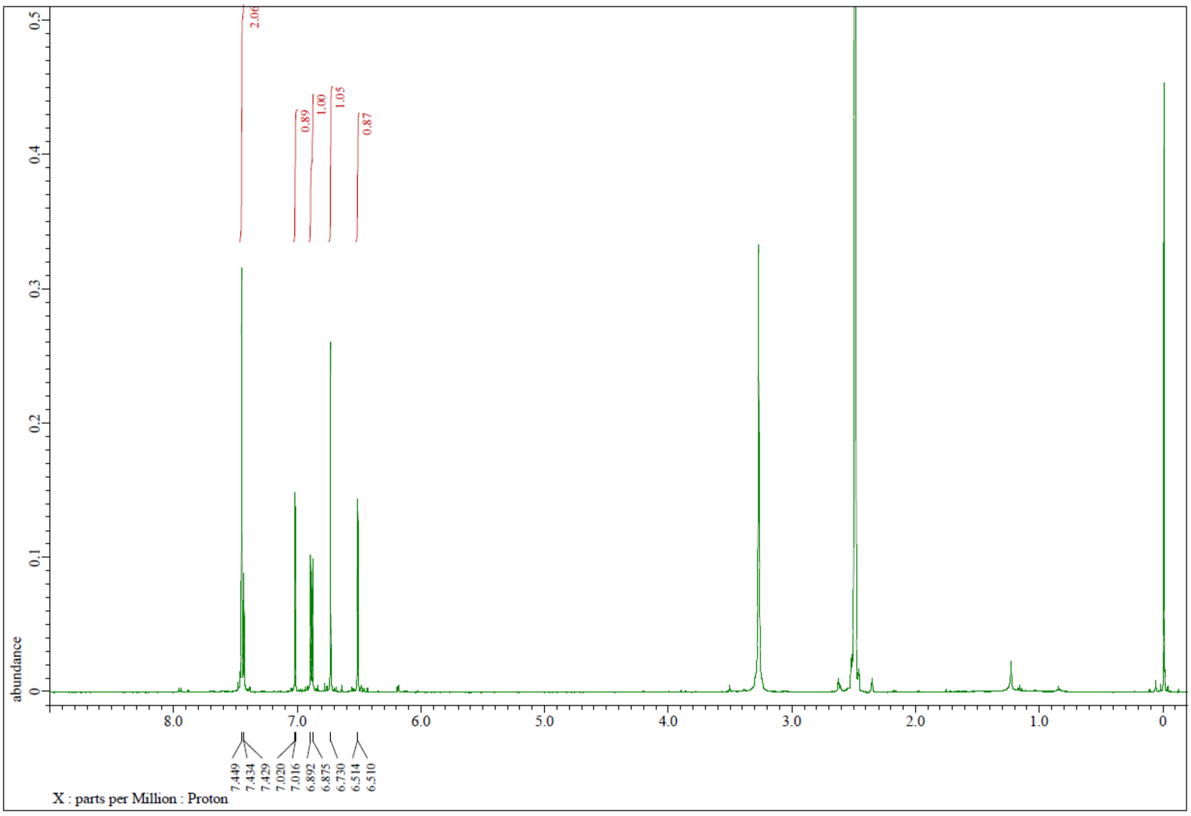

Figure S1. ^{1}H NMR Spectrum of Luteolin 7-Sulfate Potassium Salt in DMSO-d_6 (500 MHz, 35 °C)

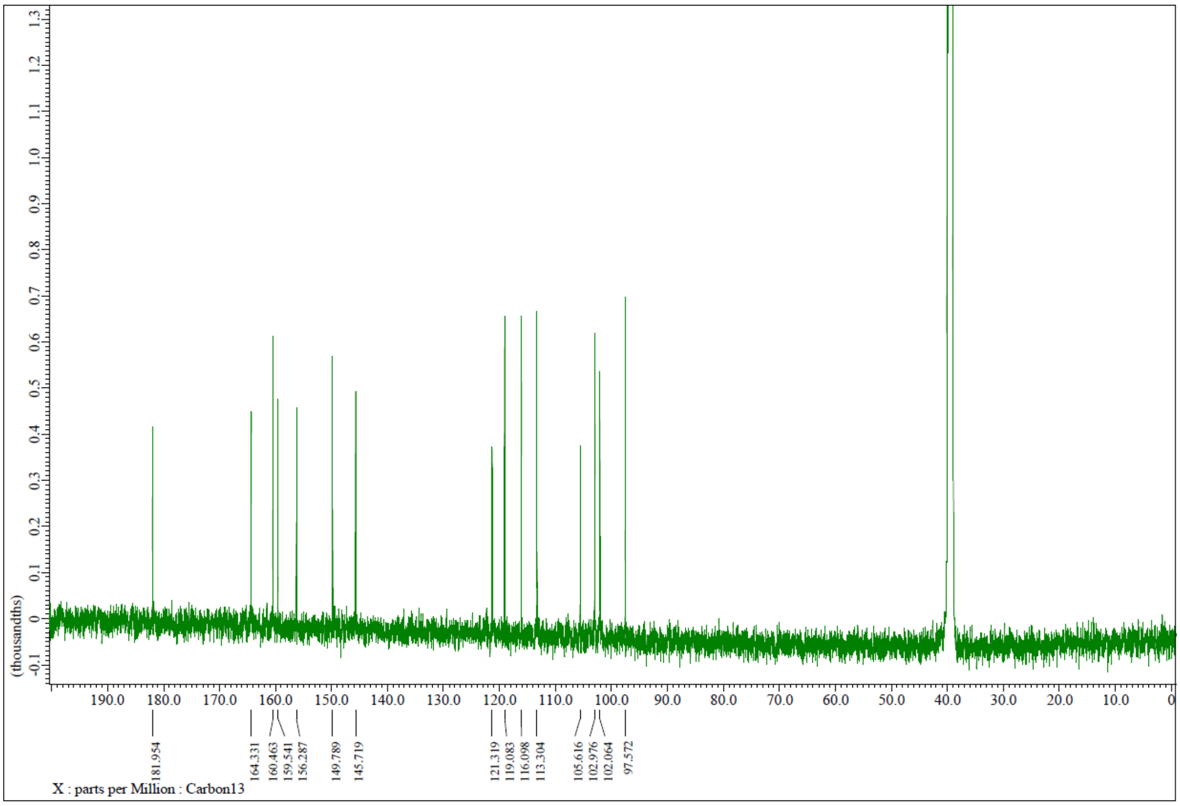

Figure S2. ^{13}C NMR Spectrum of Luteolin 7-Sulfate Potassium Salt in DMSO-d_6 (125 MHz, 35 °C)

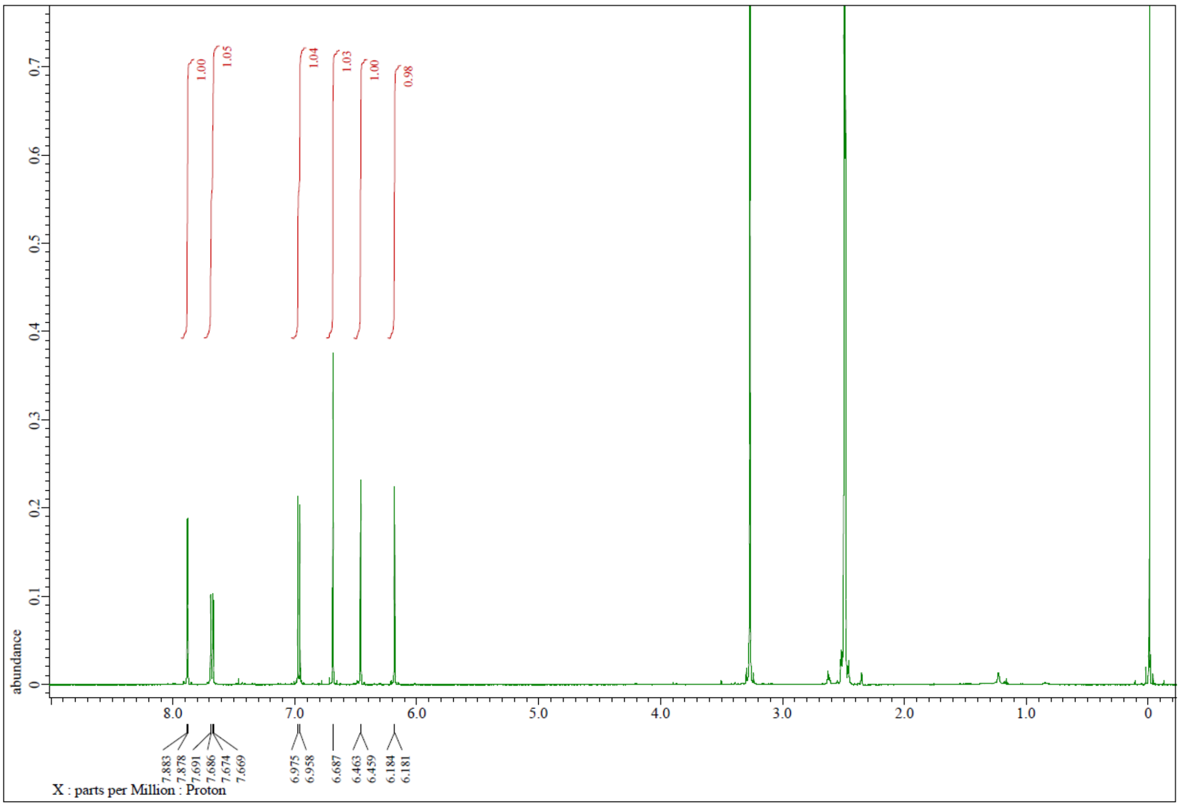

Figure S3. ^{1}H NMR Spectrum of Luteolin 3'-Sulfate Potassium Salt in DMSO-d_6 (500 MHz, 35 °C)

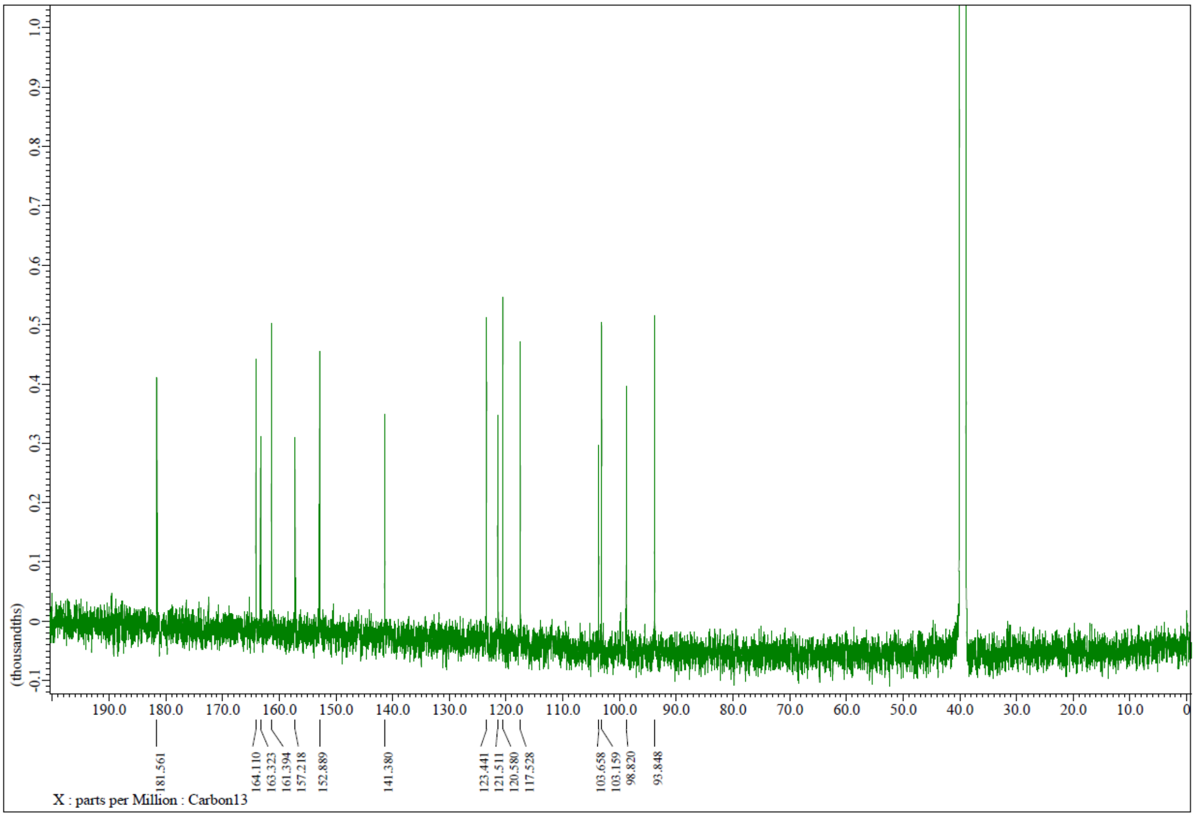

Figure S4. ^{13}C NMR Spectrum of Luteolin 3'-Sulfate Potassium Salt in DMSO-d_6 (125 MHz, 35 °C)

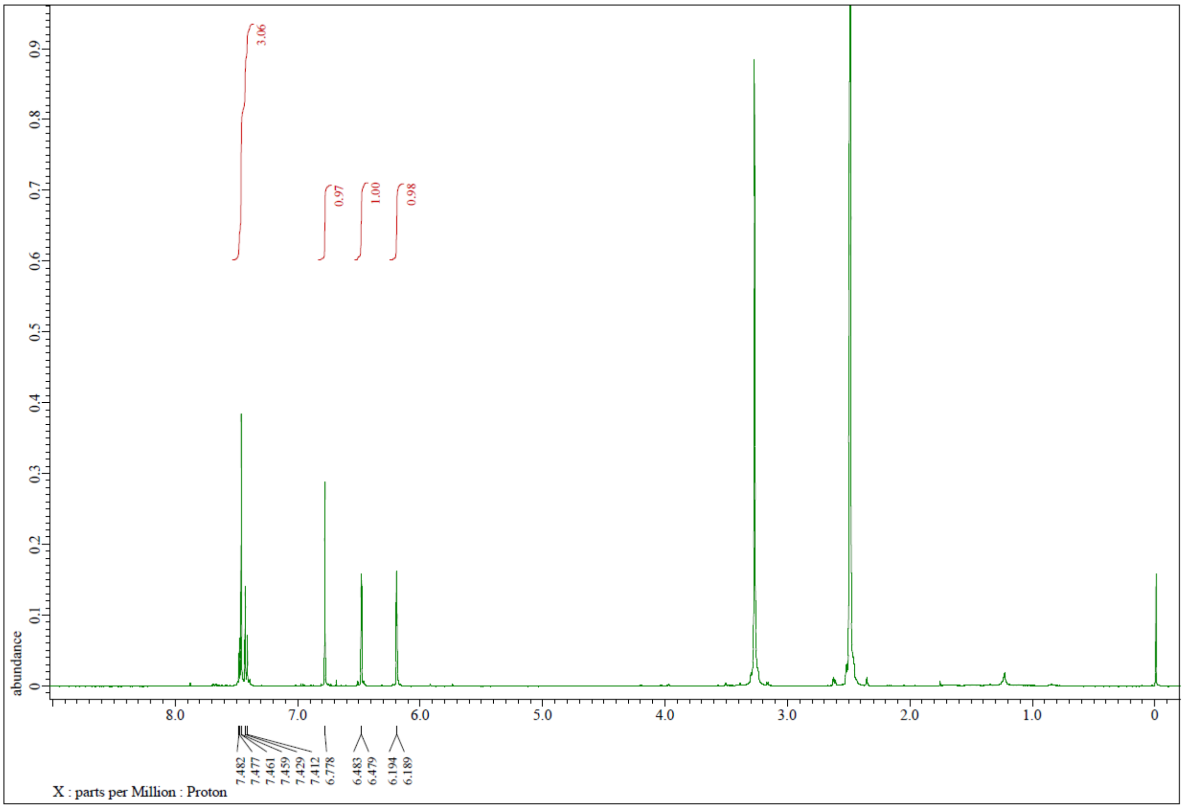

Figure S5. ^{1}H NMR Spectrum of Luteolin 4'-Sulfate Potassium Salt in DMSO-d_6 (500 MHz, 35 °C)

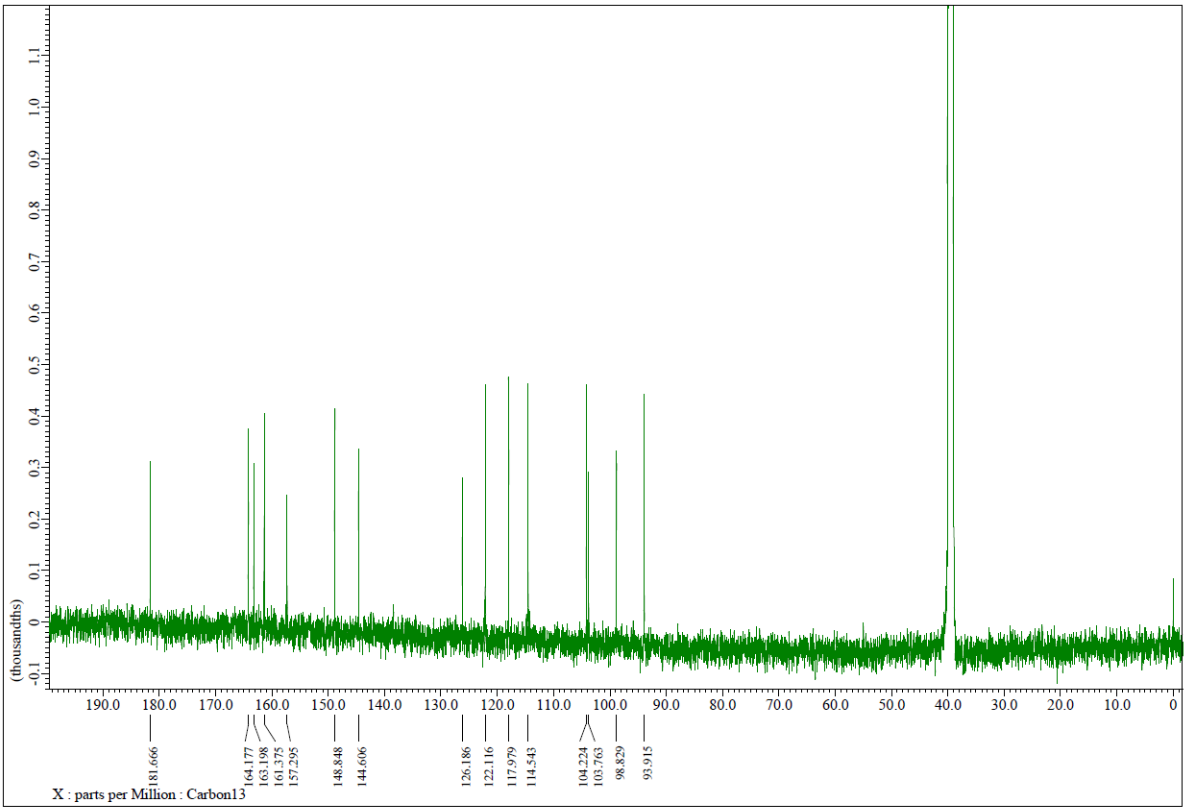

Figure S6. ^{13}C NMR Spectrum of Luteolin 4'-Sulfate Potassium Salt in DMSO-d_6 (125 MHz, 35 °C)

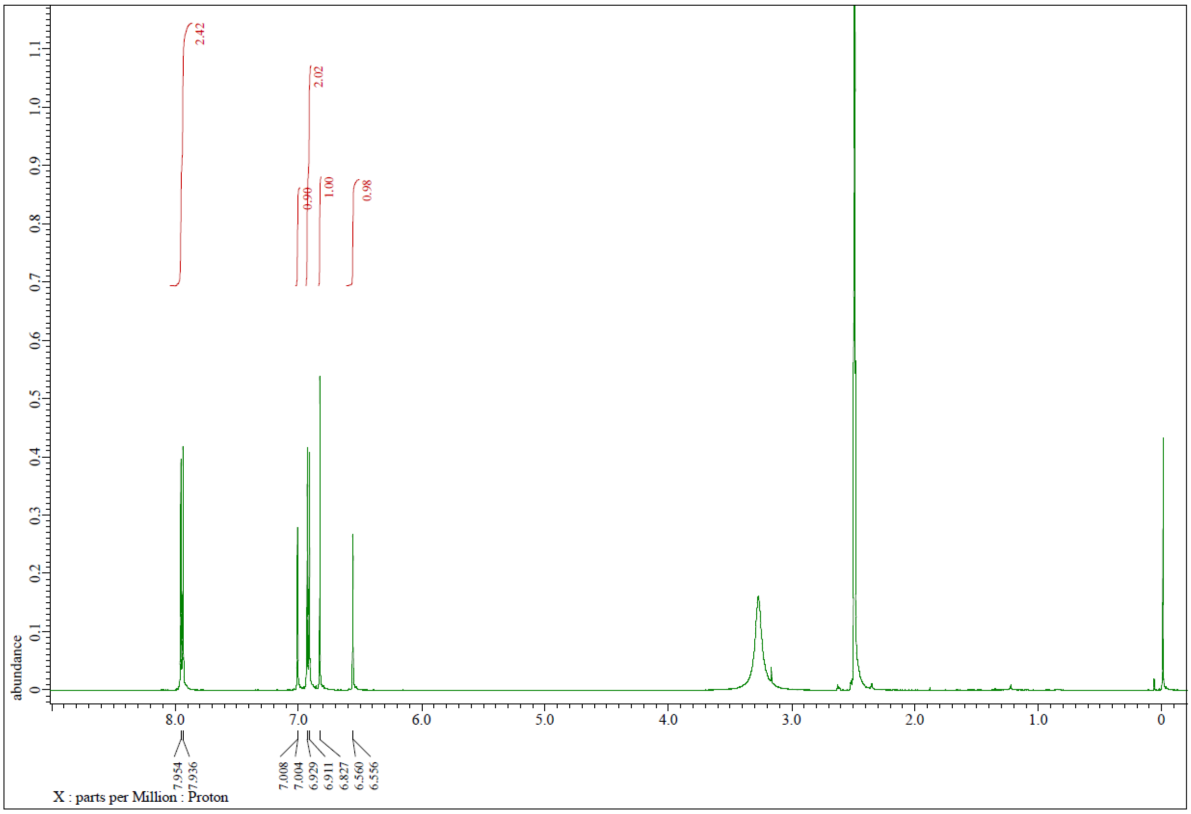

Figure S7. ^{1}H NMR Spectrum of Apigenin 7-Sulfate Potassium Salt in DMSO-d_6 (500 MHz, 35 °C)

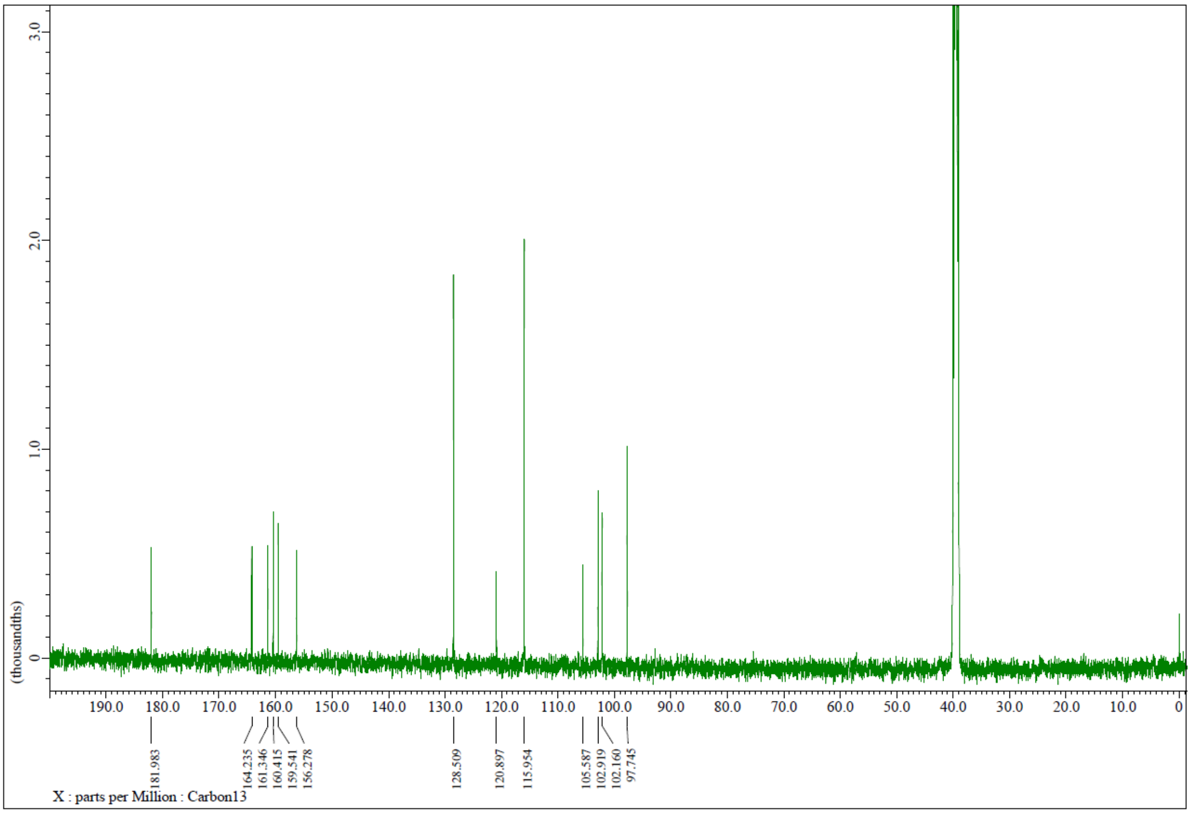

Figure S8. ^{13}C NMR Spectrum of Apigenin 7-Sulfate Potassium Salt in DMSO-d_6 (125 MHz, 35 °C)

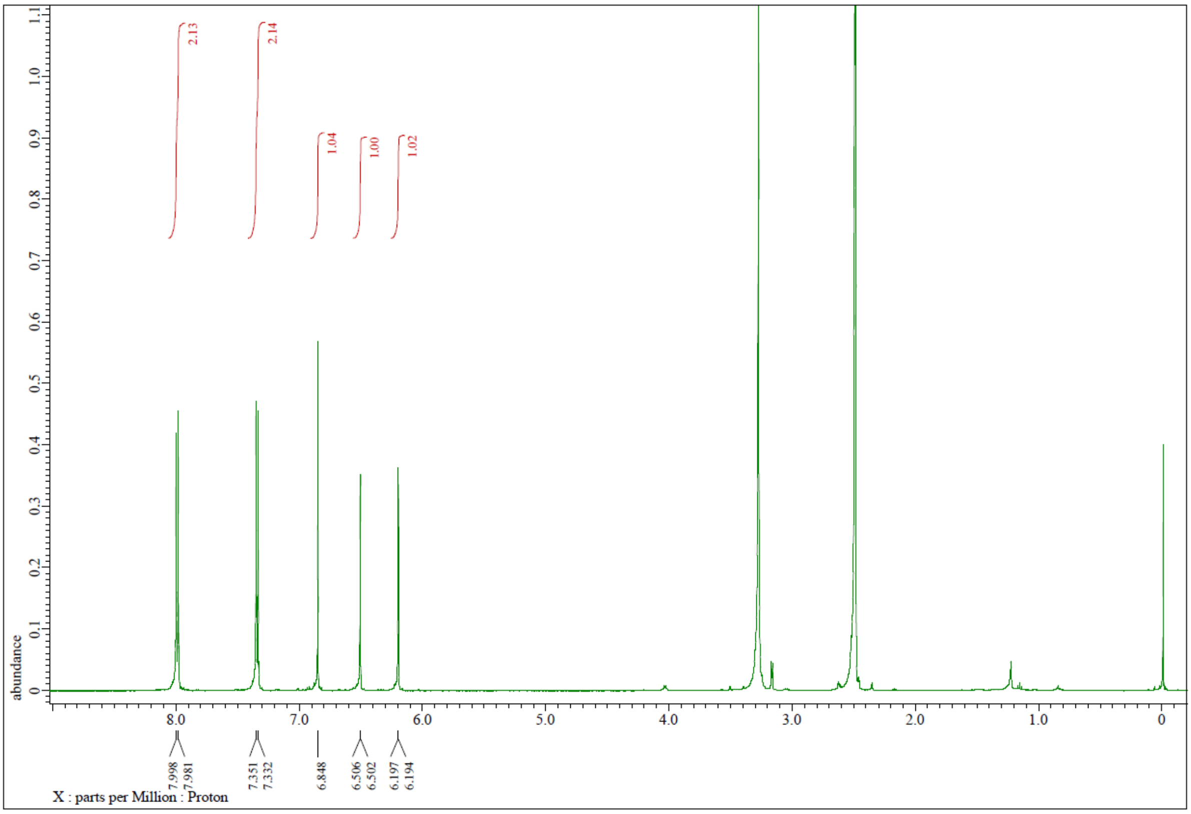

Figure S9. ^{1}H NMR Spectrum of Apigenin 4'-Sulfate Potassium Salt in DMSO-d_6 (500 MHz, 35 °C)

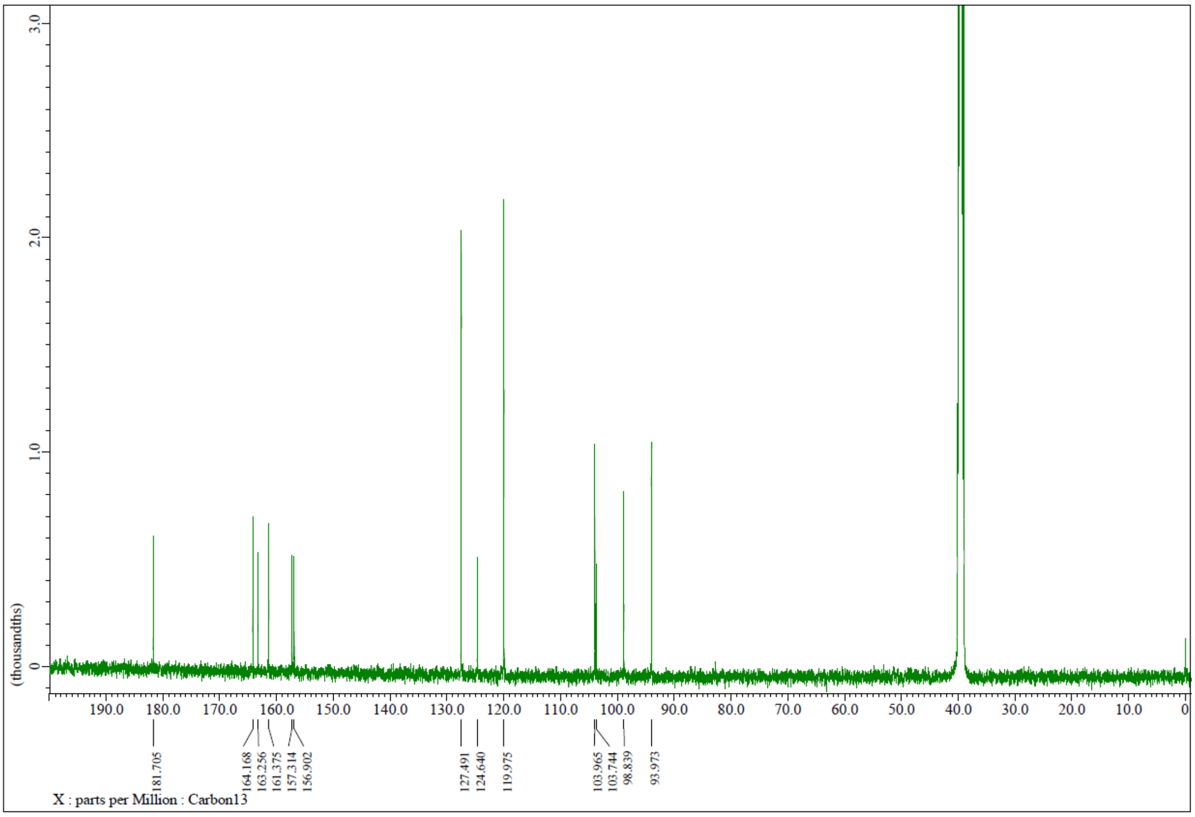

Figure S10. ^{13}C NMR Spectrum of Apigenin 4'-Sulfate Potassium Salt in DMSO-d_6 (125 MHz, 35 °C)

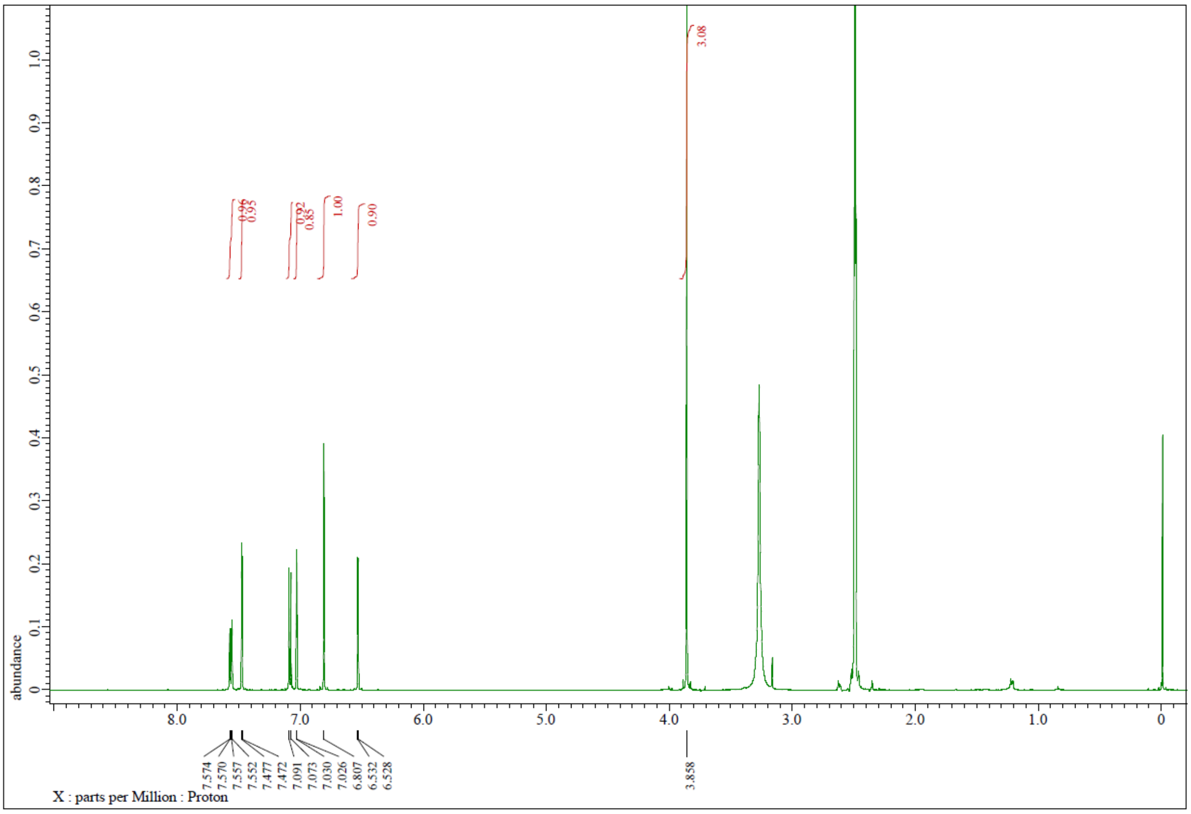

Figure S11. ^{1}H NMR Spectrum of Diosmetin 7-Sulfate Potassium Salt in DMSO-d_6 (500 MHz, 35 °C)

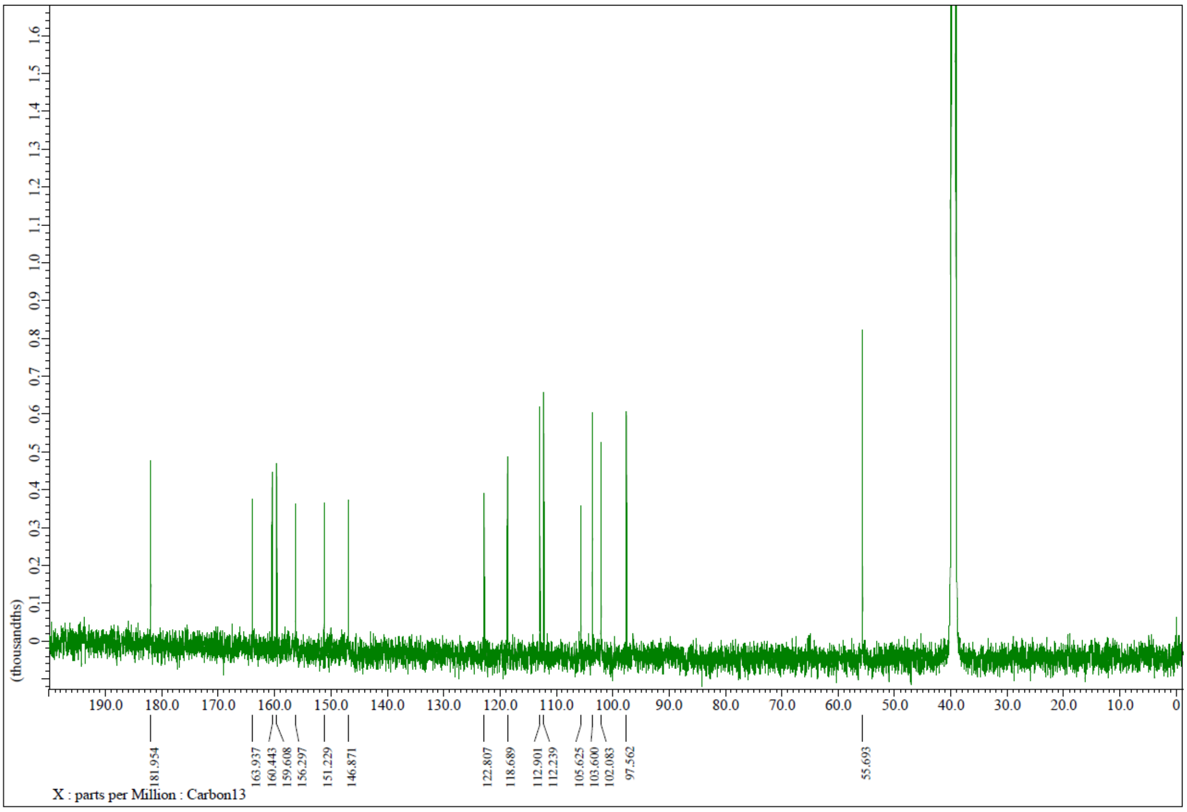

Figure S12. ^{13}C NMR Spectrum of Diosmetin 7-Sulfate Potassium Salt in DMSO-d_6 (125 MHz, 35 °C)

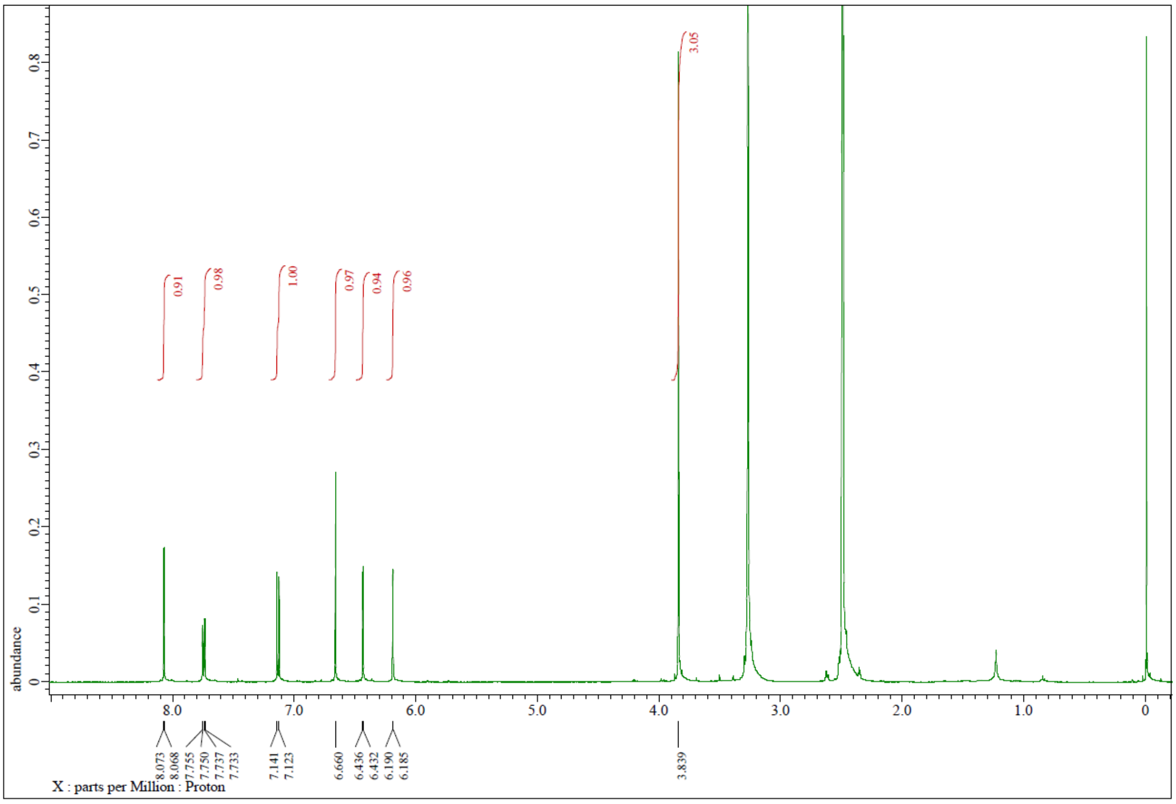

Figure S13. ^{1}H NMR Spectrum of Diosmetin 3'-Sulfate Potassium Salt in DMSO-d_6 (500 MHz, 35 °C)

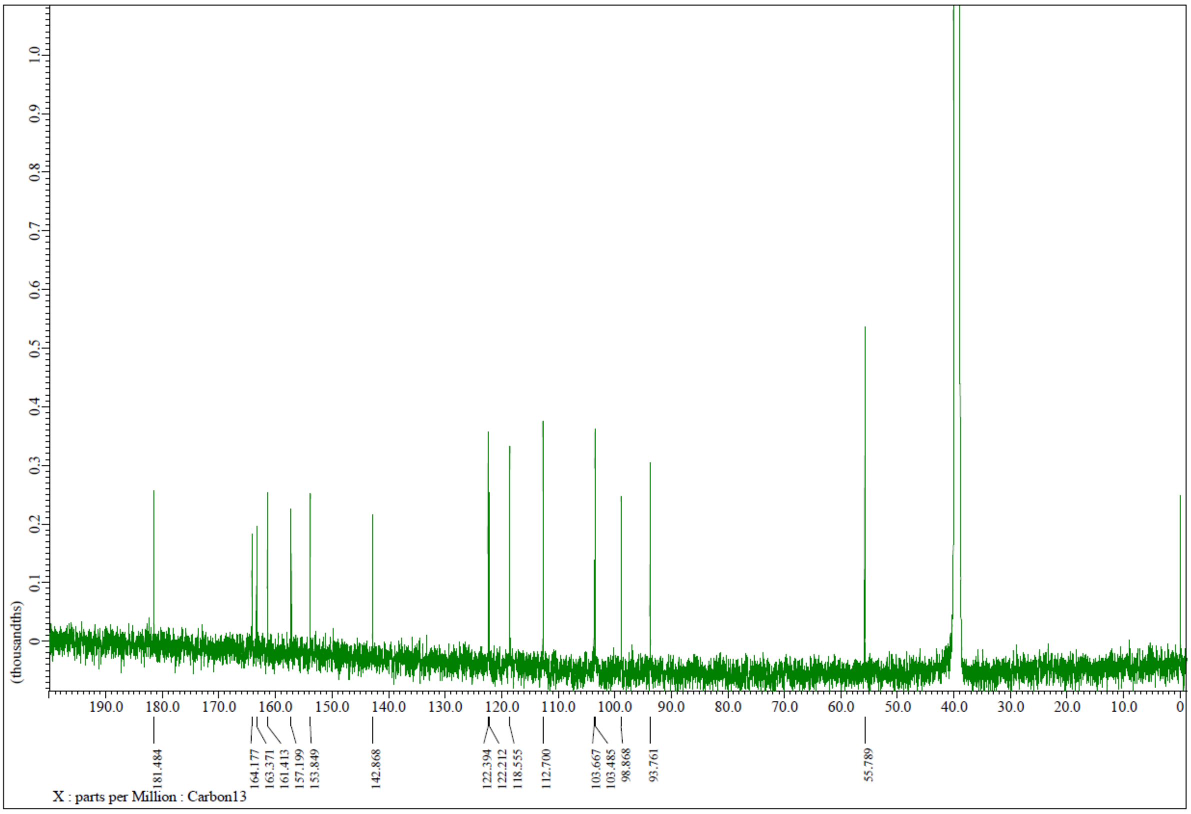

Figure S14. ^{13}C NMR Spectrum of Diosmetin 3'-Sulfate Potassium Salt in DMSO-d_6 (125 MHz, 35 °C)

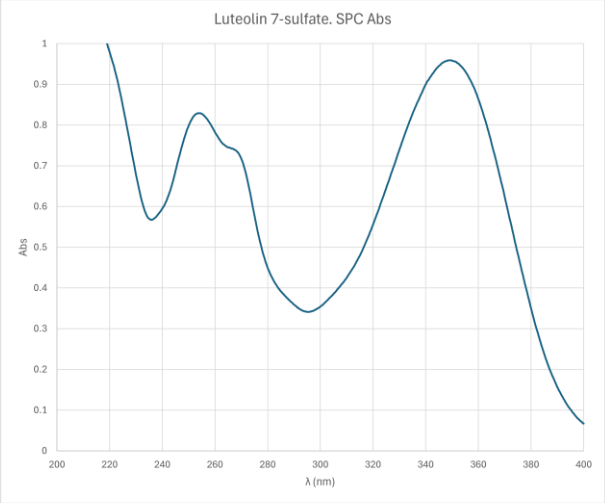

Figure S15. UV Spectrum of Luteolin 7-Sulfate Potassium Salt in MeOH

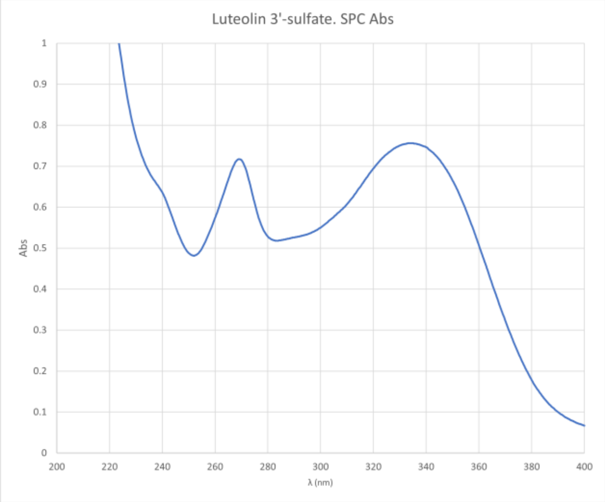

Figure S16. UV Spectrum of Luteolin 3'-Sulfate Potassium Salt in MeOH

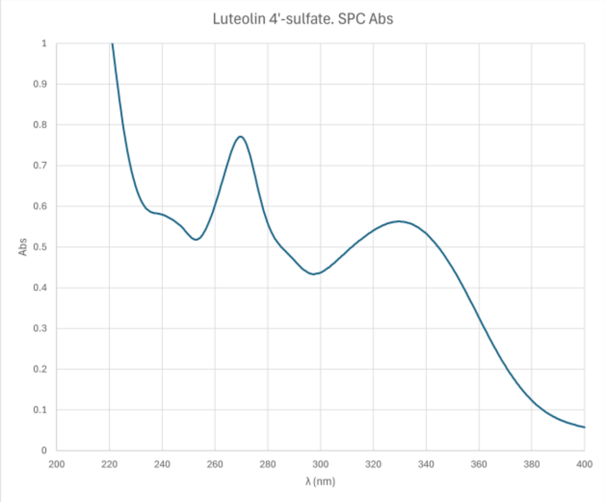

Figure S17. UV Spectrum of Luteolin 4'-Sulfate Potassium Salt in MeOH

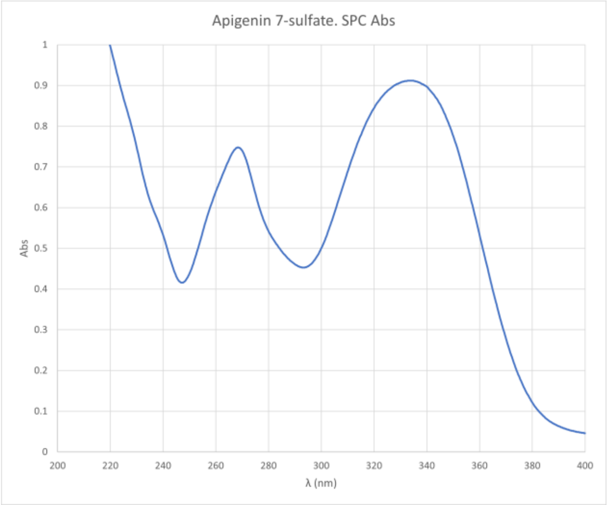

Figure S18. UV Spectrum of Apigenin 7-Sulfate Potassium Salt in MeOH

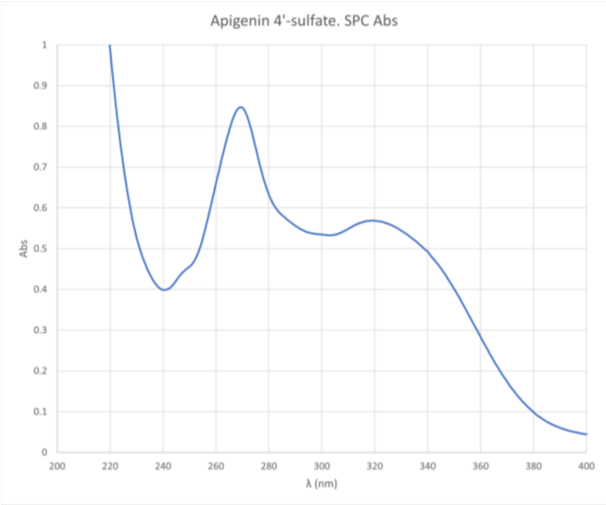

Figure S19. UV Spectrum of Apigenin 4’-Sulfate Potassium Salt in MeOH

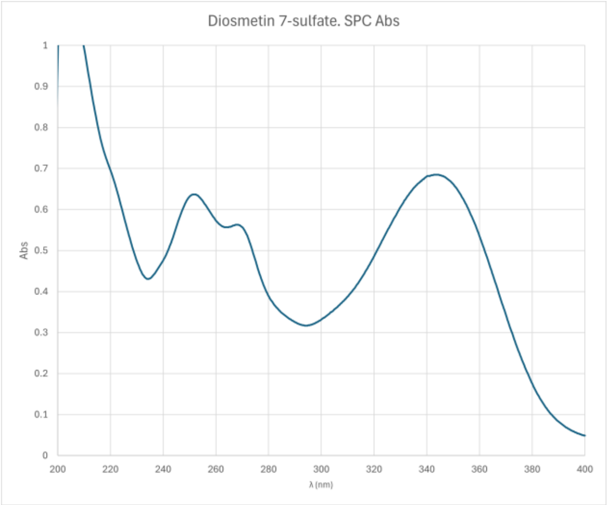

Figure S20. UV Spectrum of Diosmetin 7-Sulfate Potassium Salt in MeOH

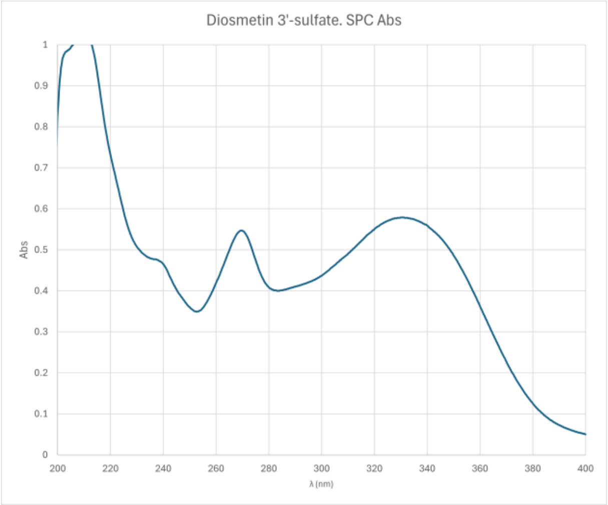

Figure S21. UV Spectrum of Diosmetin 3’-Sulfate Potassium Salt in MeOH

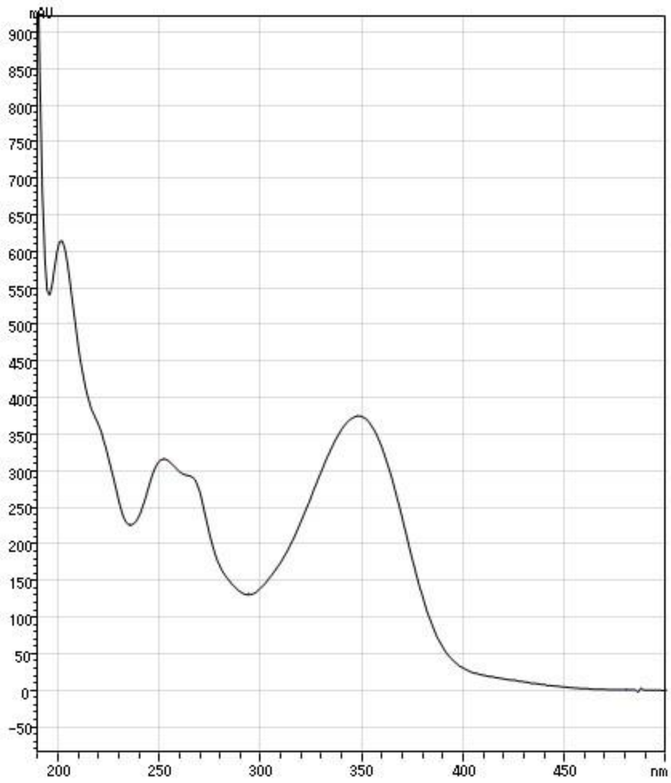

Figure S22. UV Spectrum of Luteolin 7-Sulfate measured with an HPLC-PDA detector

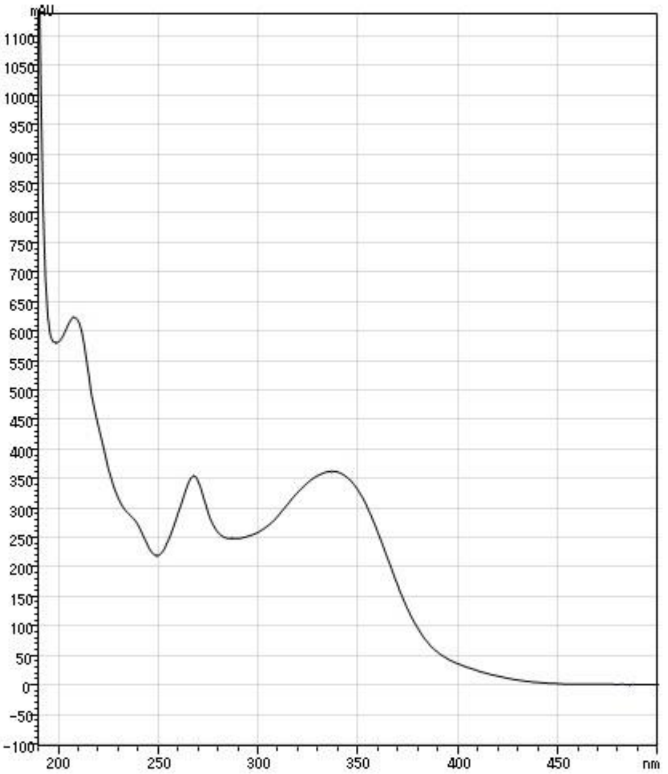

Figure S23. UV Spectrum of Luteolin 3’-Sulfate measured with an HPLC-PDA detector

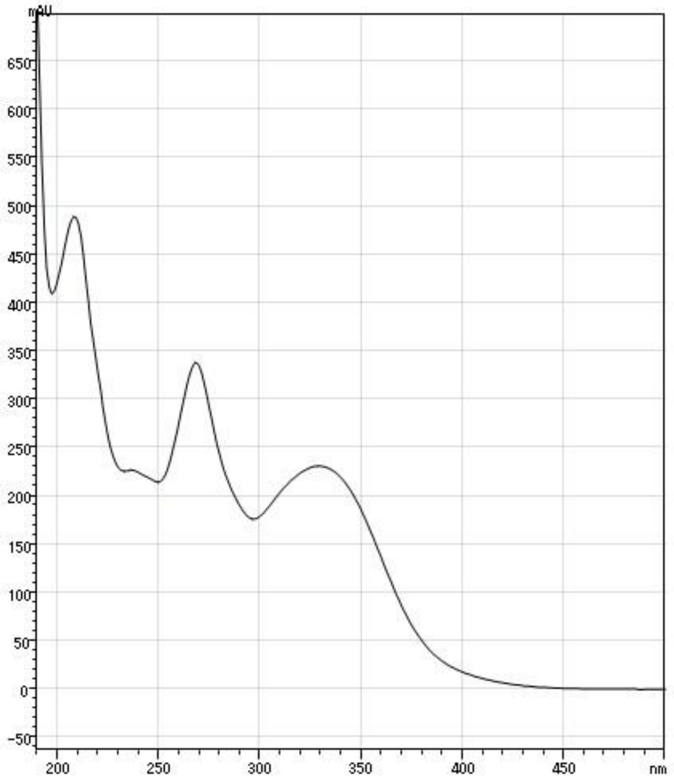

Figure S24. UV Spectrum of Luteolin 4’-Sulfate measured with an HPLC-PDA detector

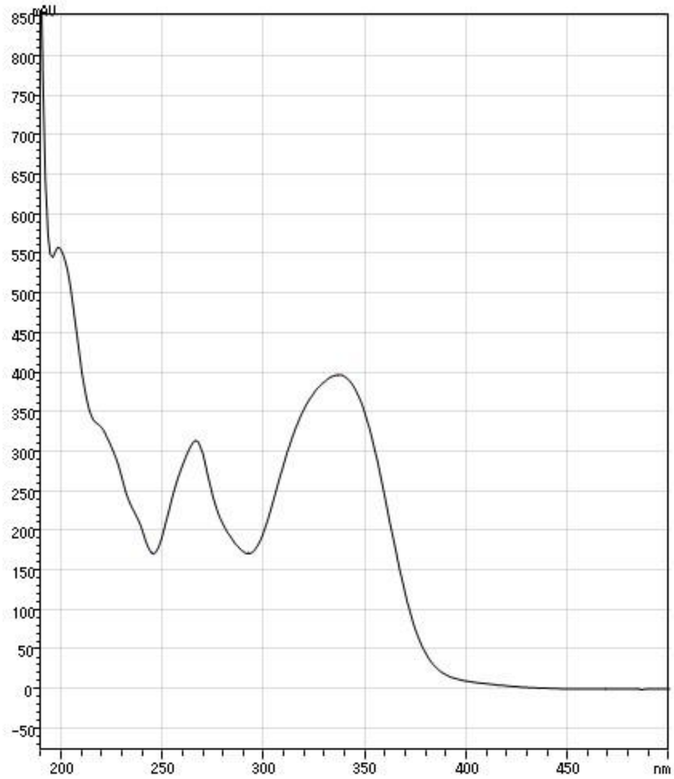

Figure S25. UV Spectrum of Apigenin 7-Sulfate measured with an HPLC-PDA detector

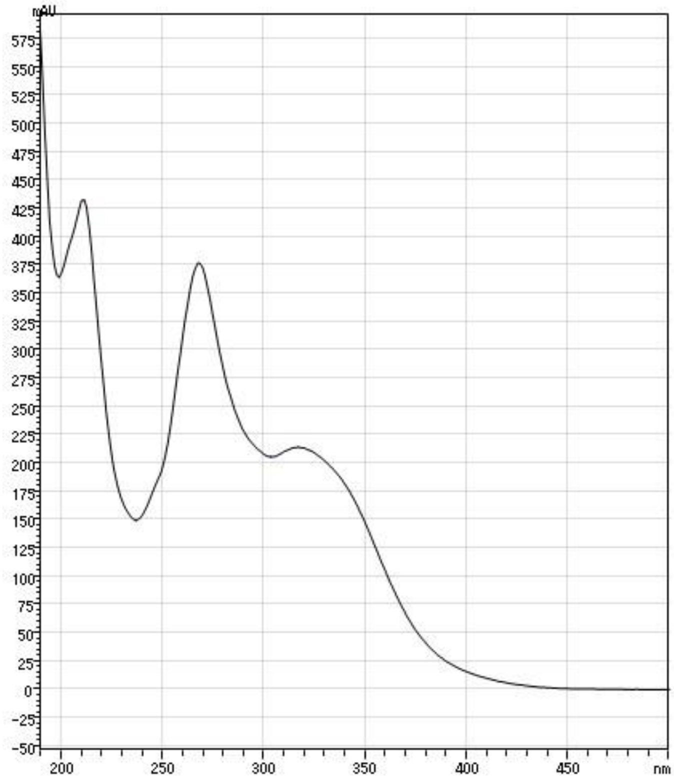

Figure S26. UV Spectrum of Apigenin 4'-Sulfate measured with an HPLC-PDA detector

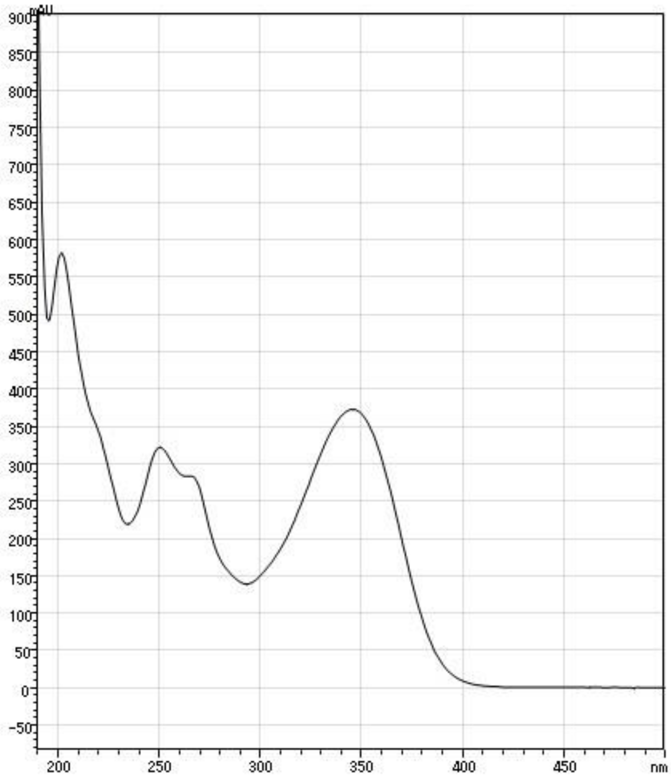

Figure S27. UV Spectrum of Diosmetin 7-Sulfate measured with an HPLC-PDA detector

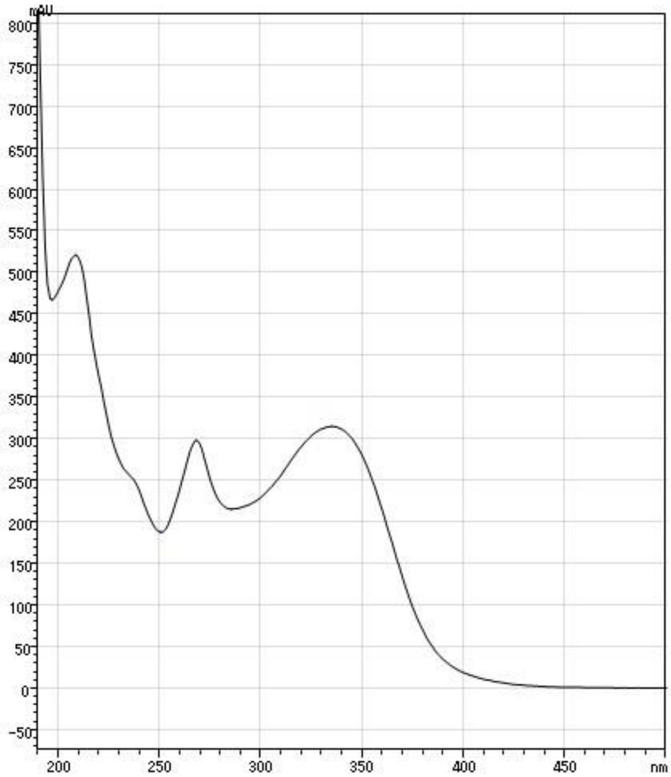

Figure S28. UV Spectrum of Diosmetin 3’-Sulfate measured with an HPLC-PDA detector

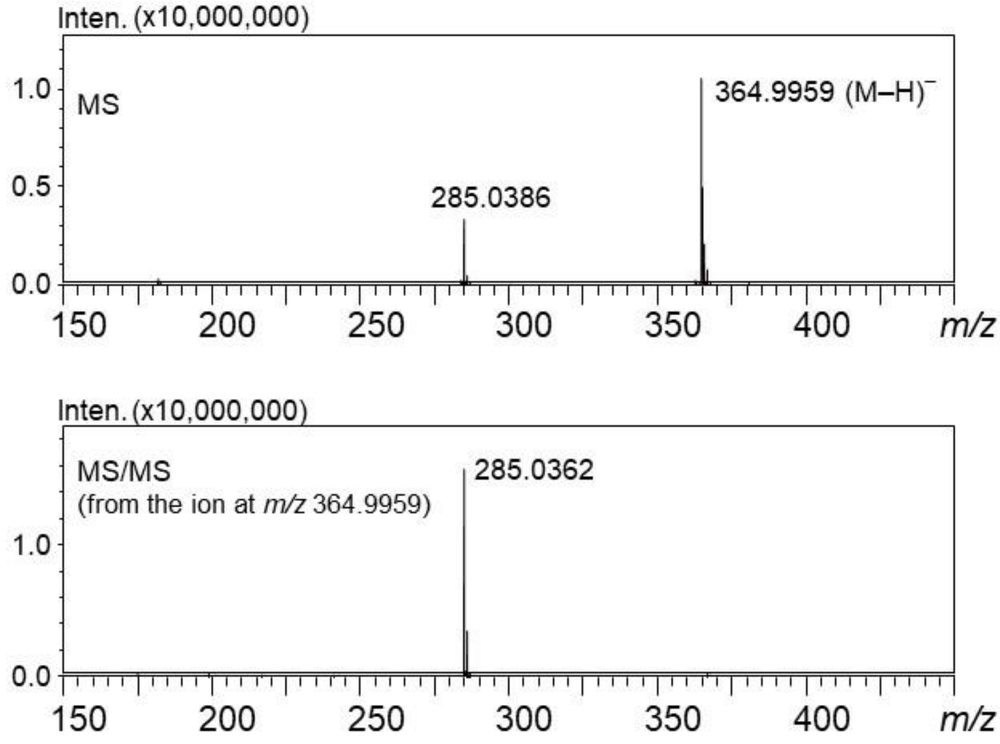

Figure S29. MS and MS/MS Spectra of Luteolin 7-Sulfate

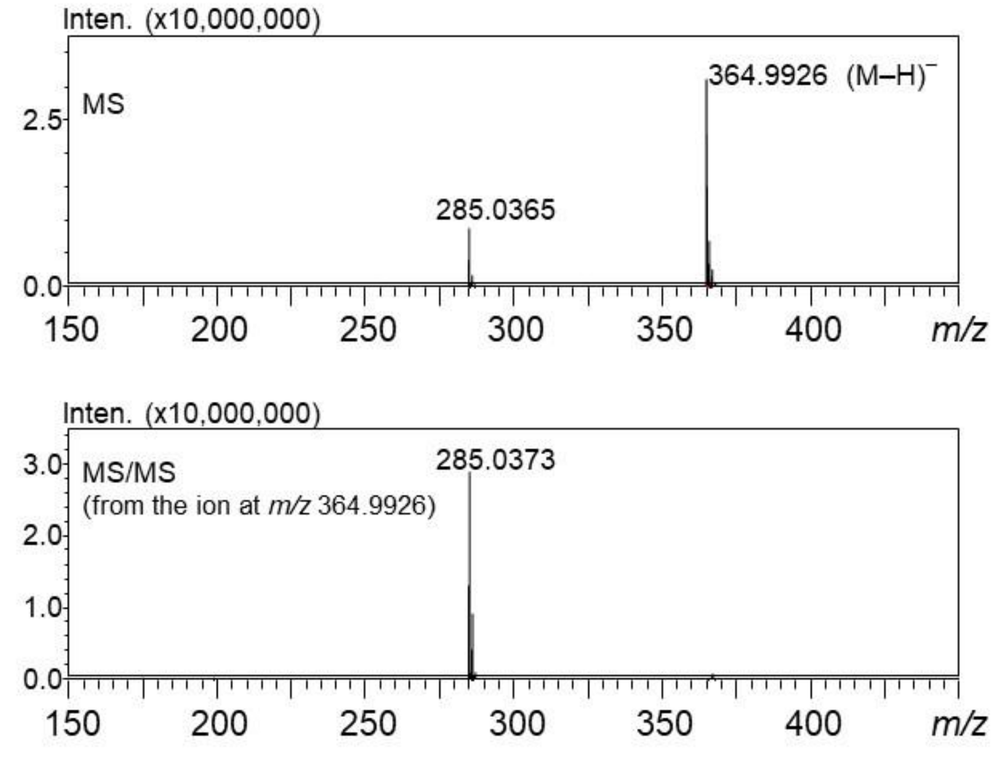

Figure S30. MS and MS/MS Spectra of Luteolin 3'-Sulfate

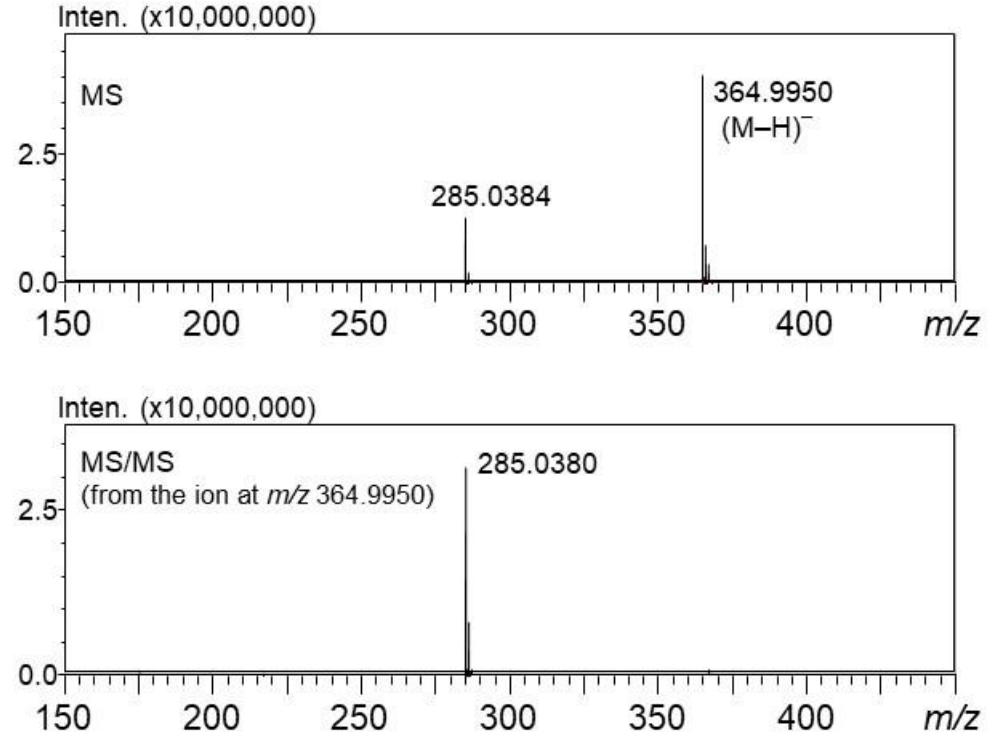

Figure S31. MS and MS/MS Spectra of Luteolin 4'-Sulfate

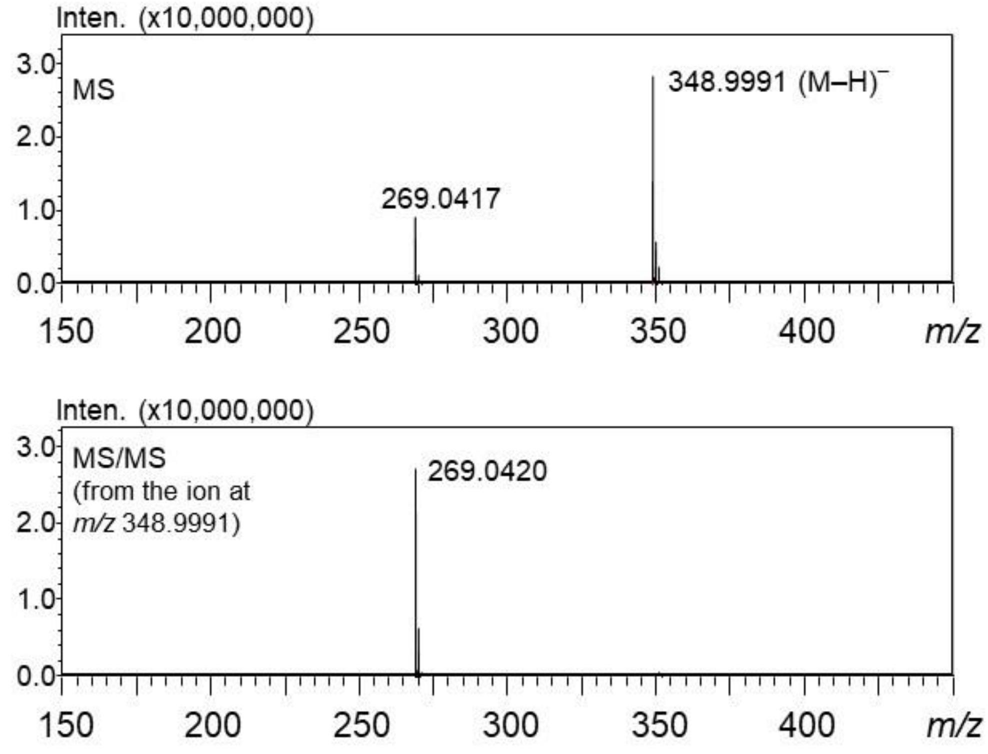

Figure S32. MS and MS/MS Spectra of Apigenin 7-Sulfate

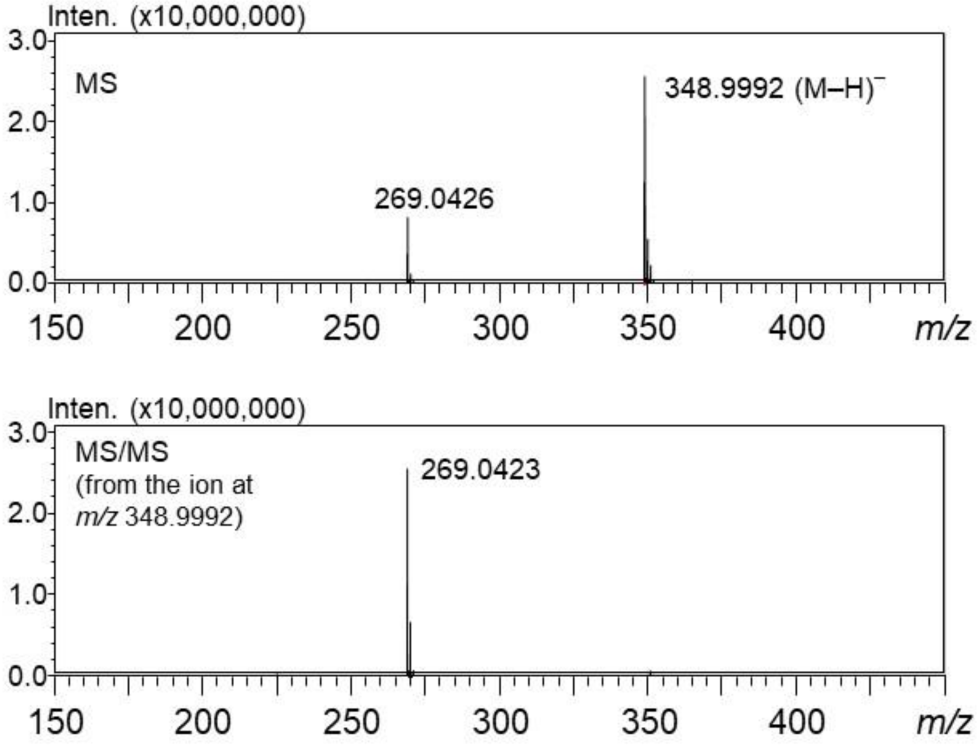

Figure S33. MS and MS/MS Spectra of Apigenin 4’-Sulfate

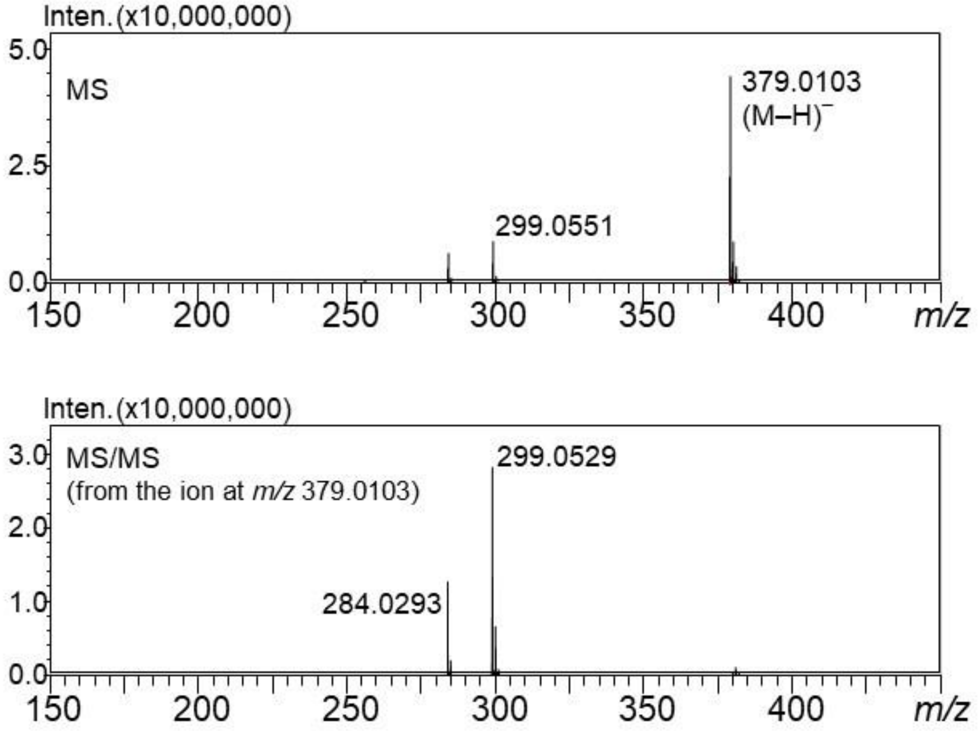

Figure S34. MS and MS/MS Spectra of Diosmetin 7-Sulfate

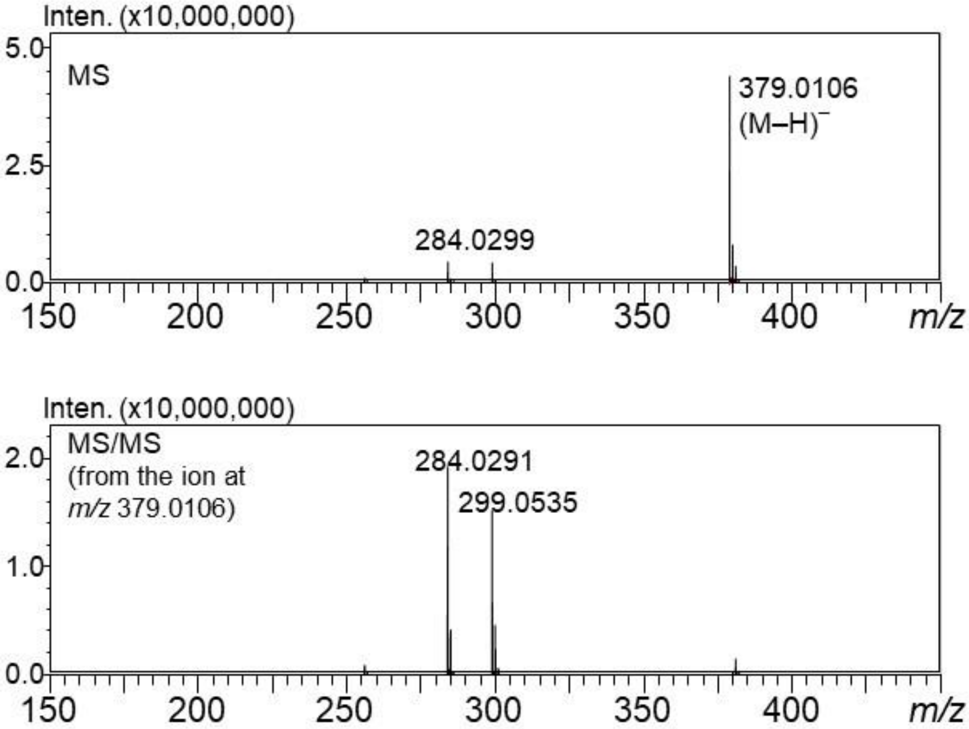

Figure S35. MS and MS/MS Spectra of Diosmetin 3'-Sulfate

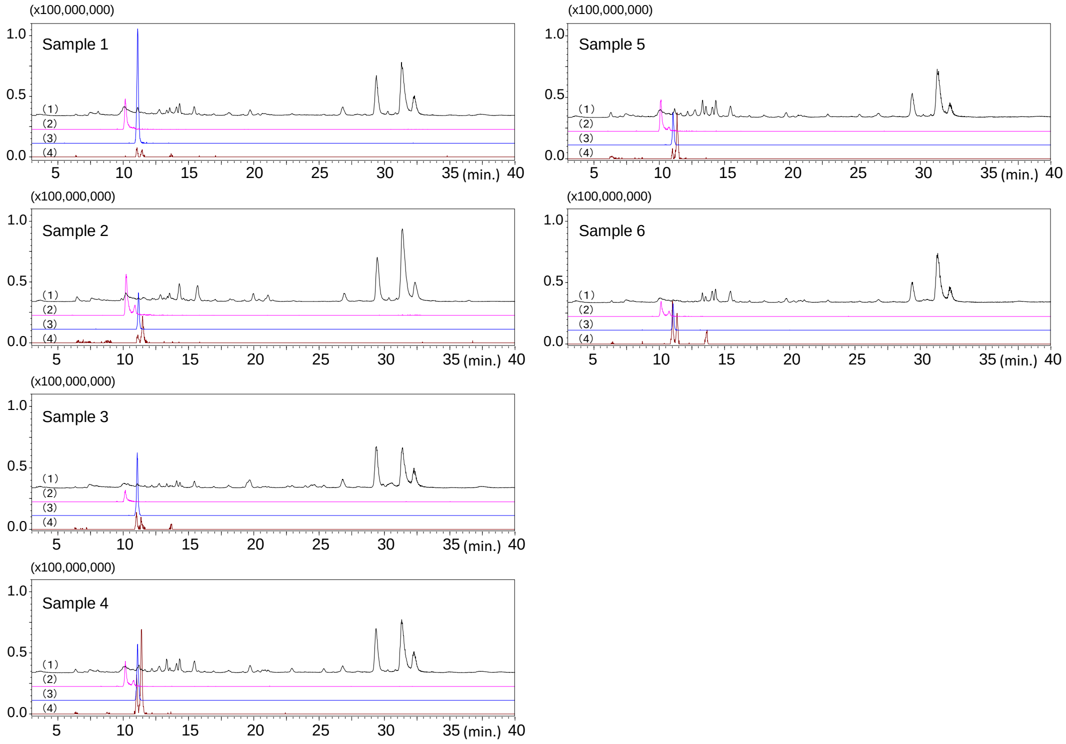

Figure S36. Liquid chromatography–mass spectrometry total-ion chromatograms and mass chromatograms of the extracts of the *E. prostrata* specimens: (1) negative-ion total-ion chromatogram, (2) $(M–H)^-$ ions of luteolin sulfate (*m/z* 364.9970, 25x magnification), (3) apigenin sulfate (*m/z* 349.0016, 40x magnification), and (4) diosmetin sulfate (*m/z* 379.0144, 100x magnification)

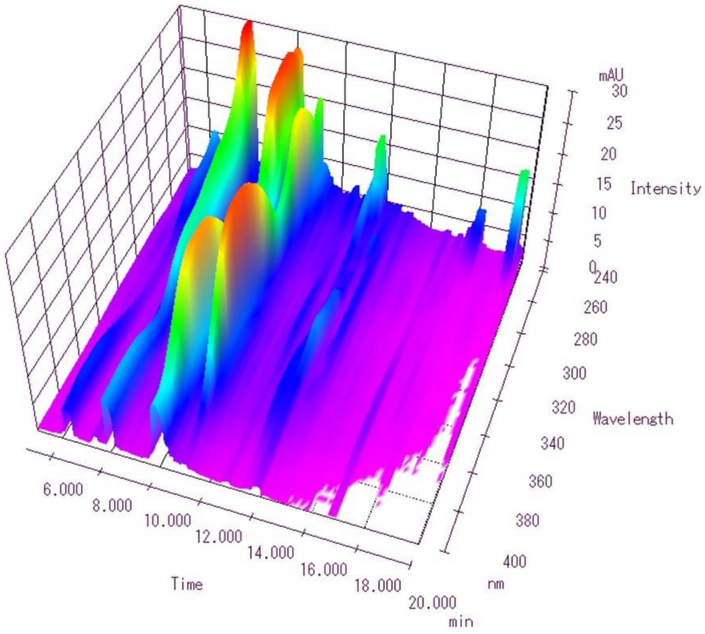

Figure S37. 3D-HPLC Chromatogram of Sample 1 (Henan Province, 2019)

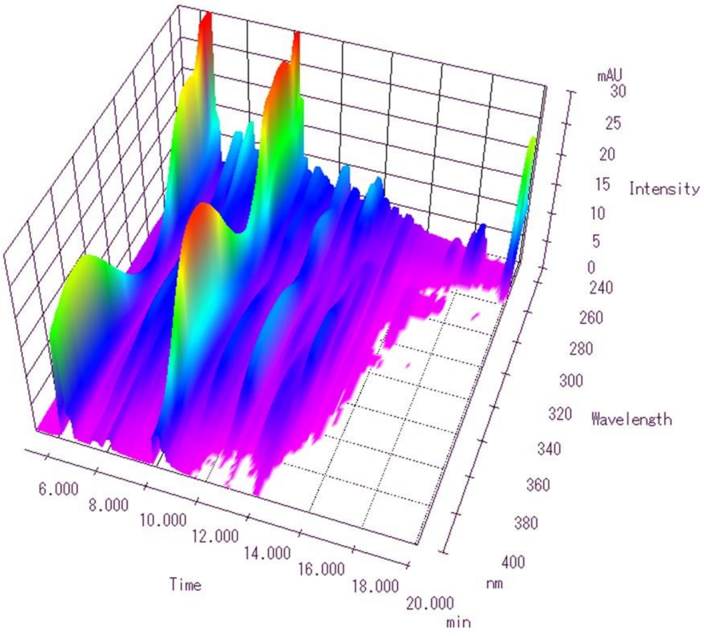

Figure S38. 3D-HPLC Chromatogram of Sample 2 (Henan Province, 2020)

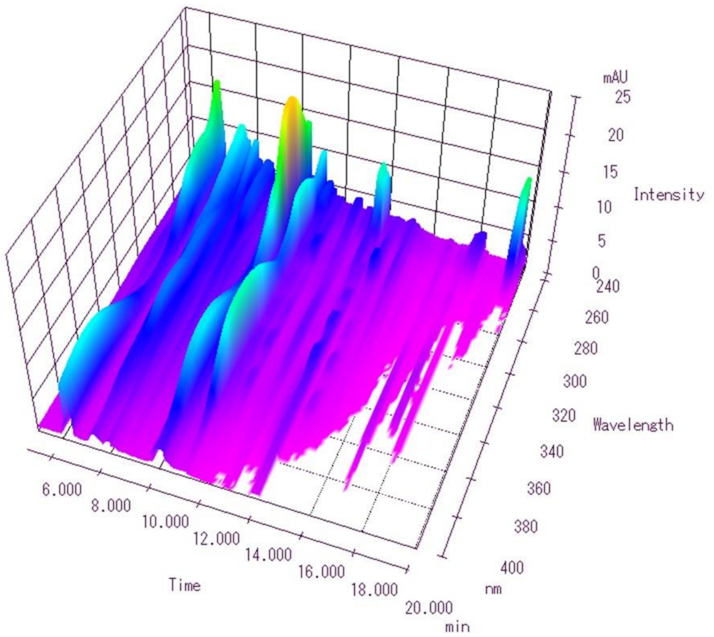

Figure S39. 3D-HPLC Chromatogram of Sample 3 (Henan Province, 2021)

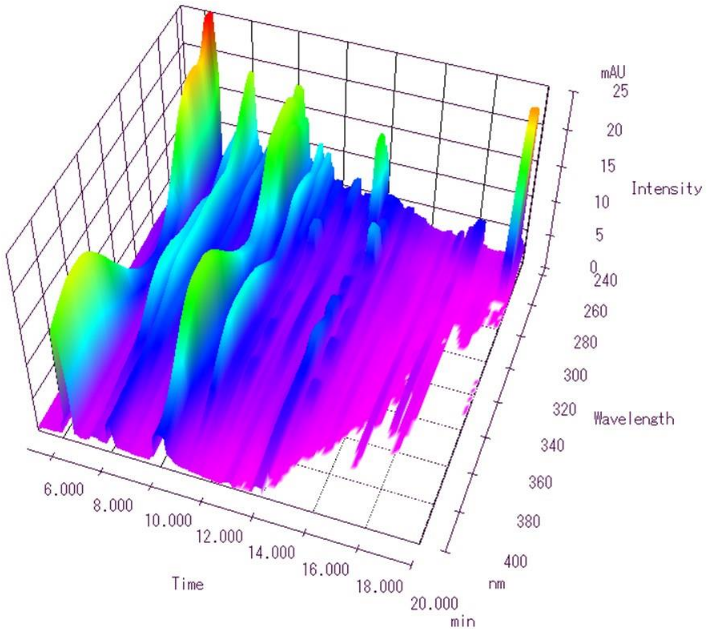

Figure S40. 3D-HPLC Chromatogram of Sample 4 (Henan Province)

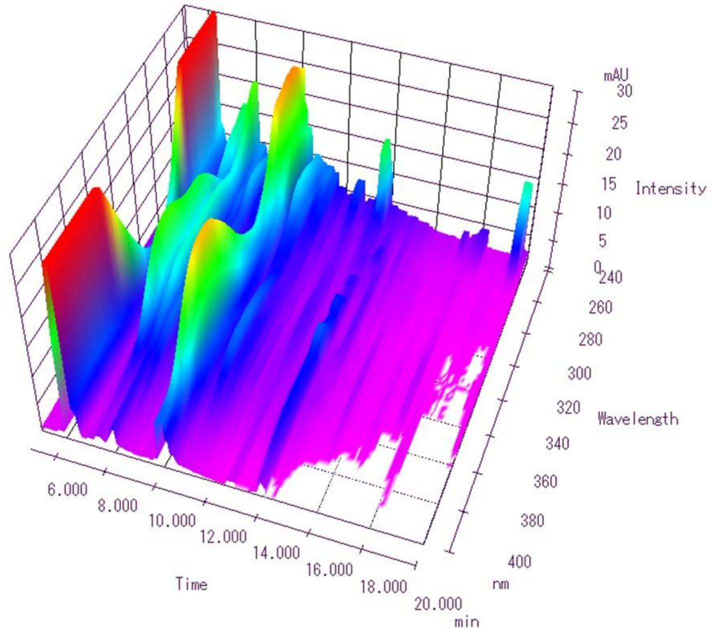

Figure S41. 3D-HPLC Chromatogram of Sample 5 (Hunan Province, 2021)

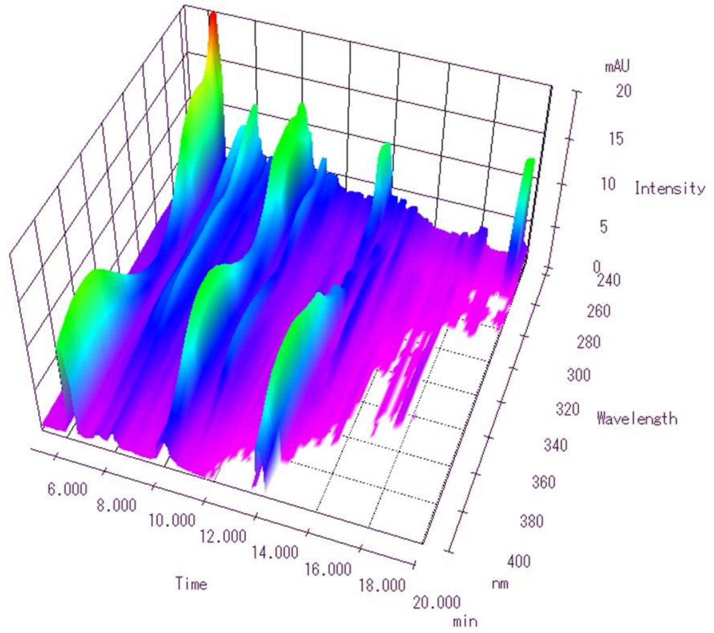

Figure S42. 3D-HPLC Chromatogram of Sample 6 (Jiangxi Province)
